# Kynurenine pathway: a possible new mechanism for exercise in the prevention and treatment of Alzheimer's disease

**DOI:** 10.3389/fnagi.2025.1617690

**Published:** 2025-10-07

**Authors:** Gangqiang Li, Shuang Li, Wenhui Zhou

**Affiliations:** ^1^Swan College, Central South University of Forestry and Technology, Changsha, China; ^2^Changde Vocational and Technical College, Changde, China

**Keywords:** exercise, Alzheimer's disease, kynurenine pathway, kynurenic acid, quinolinic acid

## Abstract

Alzheimer's disease (AD) is the most common neurodegenerative disease in clinical practice. The kynurenine pathway (KP) is a potential intersection of factors associated with the development of AD (central nervous inflammation, glutamate excitotoxicity, and tau phosphorylation, among others). Pharmacological modulators targeting KP enzymes, such as inhibitors or agonists, and their major neuroprotective metabolites are beneficial in alleviating AD progression. Exercise significantly improves AD symptoms and also impacts KP pharmacokinetics. Promoting the production of neuroprotective active metabolites by KP may be one of the central mechanisms by which exercise improves AD symptoms. This article reviews the possible role of KP in AD neurodegeneration and AD exercise prevention and treatment.

## 1 Introduction

Alzheimer's disease (AD) is a progressive neurodegenerative disease of insidious onset characterized pathologically by amyloid plaques resulting from excessive accumulation of extracellular β-amyloid (Aβ) and hyperphosphorylation of intracellular microtubule-associated protein tau leading to the formation of neurofibrillary tangles (NFTs) (Zhong et al., 2024). It is clinically characterized by generalized dementia manifestations such as memory impairment, aphasia, apraxia, agnosia, visuospatial skill impairment, executive dysfunction, and personality and behavioral changes (Safiri et al., 2024). At present, there are about 57 million AD patients worldwide, mainly concentrated in people aged 65 years and older; the prevalence of AD increases exponentially with age, and the number of AD patients is expected to increase dramatically to 152 million by 2050 (Kalyvas et al., 2024). The etiology of AD is complex and so far unknown, and it is generally accepted in the academic community that the synergistic effect of Aβ oligomers with hyperphosphorylated tau, triggering synaptic loss, neuronal death, and abnormal glial activation, and disrupting hippocampal-cortical neural network synchrony, leading to multilevel systemic decline in memory coding, integration, and executive function is the core mechanism of the pathological process of AD (Ramachandran et al., 2021; Reddi Sree et al., 2025; Zádori et al., 2018). Existing clinical treatments for AD mainly use single-target intervention strategies such as anti-amyloid, neuroprotection, or synaptic repair, which temporarily alleviate the progression of the disease, but have limited effects and are often accompanied by serious adverse effects (Cummings et al., 2021). Faced with this severe situation, there is an urgent need for in-depth study to reveal its pathological process and find new strategies or methods to delay the AD process and prevent the occurrence of AD.

Tryptophan (TRP)—Kynurenine metabolic pathway (KP) produces metabolites with neuroactive and anti-inflammatory properties that play an important role in maintaining neurohomeostasis and disease progression (Xue et al., 2023). KP is considered to be a key inflammatory regulatory hub in the etiology of AD, and abnormal changes in its metabolic enzyme activity and metabolite content can significantly affect the oligomerization and toxicity of Aβ, and regulate the intensity and characteristics of the inflammatory response, which in turn leads to neuronal dysfunction and triggers AD neurodegeneration, so KP abnormalities are regarded as part of the pathogenesis of AD (Savonije and Weaver, 2023). This idea has been demonstrated in both animal models and clinical observations (Chatterjee et al., 2019; Parker et al., 2023; Wu et al., 2013). Recent findings have shown that pharmacological modulators by targeting KP enzymes may serve as an effective neuroprotective strategy in AD (Martins et al., 2023; Yu et al., 2015). Exercise, as a non-pharmacological intervention for AD, synergistically regulates neurotransmitter homeostasis and enhances neuroplasticity through multiple effects such as neuroprotection, anti-inflammation and anti-oxidative stress (Li et al., 2024); its unique mechanism of action in delaying the pathological progression of AD has attracted much attention, and dissecting the potential molecular mechanisms behind exercise in relieving AD symptoms is a key point in the prevention and treatment of AD. Exercise regulates KP-related enzyme expression and associated metabolite levels to maintain KP homeostasis (Rangel et al., 2025) and is beneficial in neurodegenerative diseases, including AD (Joisten et al., 2020). Promoting KP-prone neuroprotective active metabolite branching may be one of the central mechanisms by which exercise improves AD symptoms. In this review, we systematically review the role and mechanism of KP in AD neurodegeneration and AD exercise prevention and treatment, in order to provide a theoretical basis and new ideas for the study of potential molecular mechanisms of exercise intervention in relieving AD symptoms and targeted intervention.

## 2 KP

**Figure 1:**
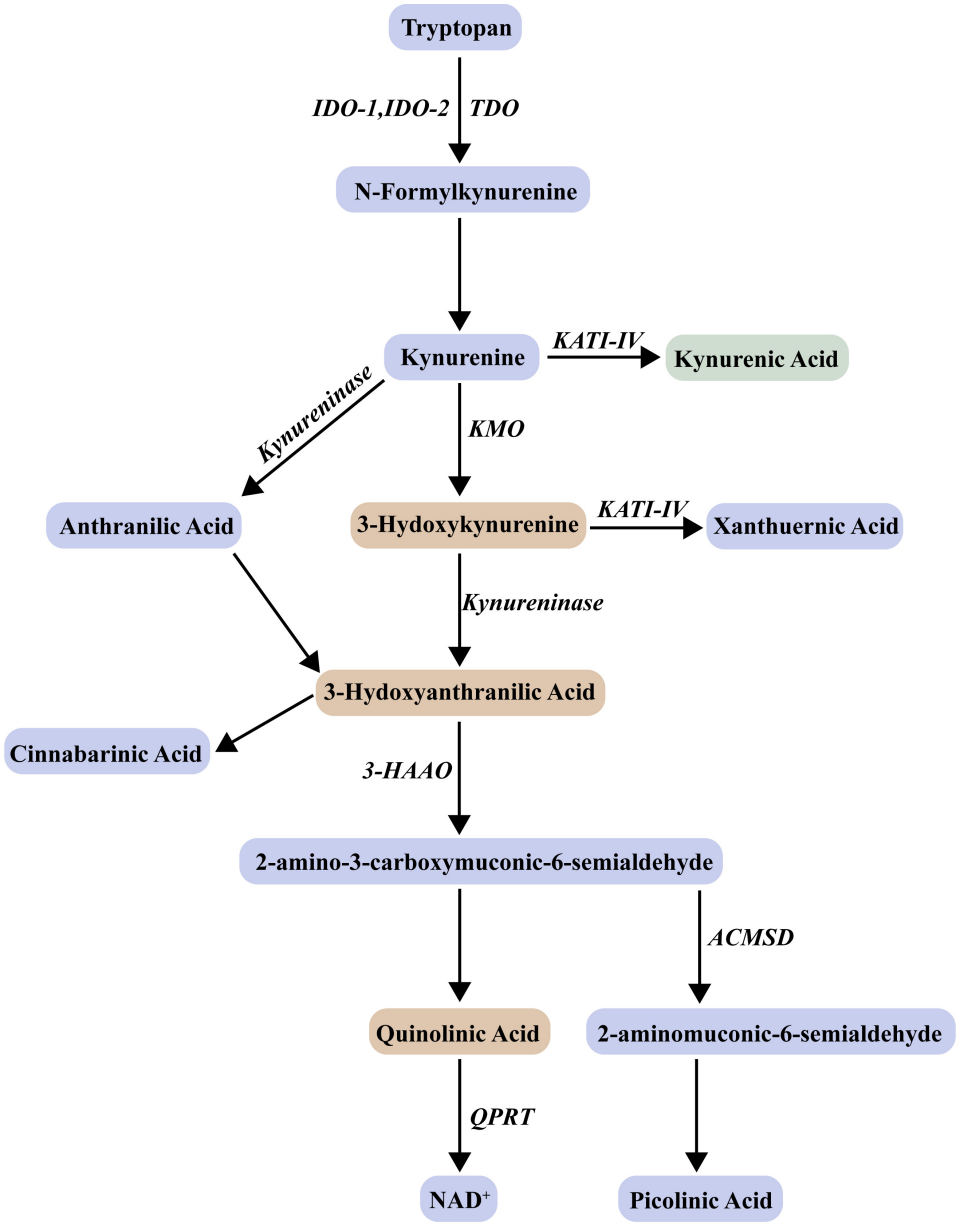
Tryptophan-kynurenine metabolic pathway. IDO, indoleamine 2,3-dioxygenase; TDO, tryptophan 2,3-dioxygenase; KATs, kynurenine aminotransferase; KMO, kynurenine-3-monooxygenase; 3-HAO, 3-hydroxyanthranilic acid 3,4-dioxygenase; ACMSD, α-amino-β-carboxymucoconate-ε-semialdehyde decarboxylase; QPRT, quinolinic acid phosphoribosyl transferase; NAD^+^, nicotinamide adenine dinucleotide.

KP is the main catabolic pathway of the essential amino acid TRP in the body, and nearly 95% of Trp is enzymatically degraded and metabolized through this pathway to generate a series of kynurenine derivatives (Figure 1) (Walczak et al., 2023). First, TRP is converted to N-formylkynurenine (N-fKYN) by three oxygen-reducing rate-limiting enzymes, indoleamine 2,3-dioxygenase (IDO) 1,2 or tryptophan-2,3-dioxygenase (TDO) (Wang et al., 2025). N-Formyl kynurenine is subsequently degraded to the first intermediate stable product, kynurenine (KYN). KP enzymes and their metabolites are widely present in different mammalian tissues and cells, and their expression is mainly regulated by mediators of the immune system (Cortés Malagón et al., 2024). IDO (inducible high expression) and TDO function similarity, but have different substrate specificity, tissue distribution and expression regulation. Physiologically, TRP mediates KP basal metabolism mainly by TDO located in liver and neuronal cells. However, IDO, which is widely expressed in multiple organs throughout the body, has low physiological activity, but when the body is under immune activation or chronic stress, IDO is overexpressed at the transcriptional level induced by the main inducers interferon-γ (IFN-γ) and specific inflammatory stimulating factors such as lipopolysaccharide (LPS) tumor necrosis factor-α (TNF-α), toll-like receptor (TLR), and CLA4, resulting in increased KYN levels in peripheral and central nervous tissues (Liang et al., 2022). Next, KYN, which is located at the KP metabolic center node, generates neurotoxic or neuroprotective products through metabolism in two major branches, respectively. In microglia and macrophages, KYN metabolizes KYN to 3-hydroxykynurenine (3-HK), 3-hydroxyanthranilic acid (3-HAA), and QUIN mainly by kynurenine-3-monooxygenase (KMO) and 3-hydroxyanthranilate-3,4-dioxygenase (HAAO) (Chen and Geng, 2023). KYN is considered a potential blood biomarker associated with cognitive impairment (Vints et al., 2024). Over 60% of peripheral KYN can be transported across the blood-brain barrier (BBB) by large neutral amino acid transporter 1 (LAT-1) as well as organic anion transporters (OATs) 1 and 3 (Mor et al., 2021). Its precursor TRP is also transported into the central nervous system (CNS), but KYNA and QUIN are unable to cross the BBB (Török et al., 2020). Under pathological conditions, QUIN acts synergistically through multiple pathways to lead to neuronal dysfunction and/or death. First, QUIN acts as an agonist of ionotropic glutamate receptors (iGluR) and α7 nicotinic acetylcholine receptors (α7nAChR), inducing excitotoxicity in neurons to promote neurodegeneration (Platten et al., 2019). In addition, QUIN not only directly stimulates glutamate (Glu) release from the presynaptic membrane of neurons, but also increases Ca^2+^ influx by inhibiting Glu reuptake by astrocytes and decreasing glutamine synthase activity in a dose-dependent manner, resulting in Glu accumulation in the synaptic cleft of neurons to form an abnormal excitotoxic microenvironment, further activating N-methyl-D-aspartate receptor (NMDAR), interfering with the homeostasis of the Glu-glutamate-γ-aminobutyric acid cycle between neurons and glia, and aggravating Glu excitotoxic effects (Tavares et al., 2002; Ting et al., 2009; Yang et al., 2024). More critically, QUIN can also perturb cellular phosphorylation homeostasis by continuously activating NMDAR, induce pathological phosphorylation of tau and neurofibrillary tangle (NFT) formation while (Rahman et al., 2009), increase abnormal phosphorylation of cytoskeletal components such as neurofilament and astrocyte GFAP, ultimately leading to synaptic structure destruction and neuronal degeneration (Guillemin, 2012; Lugo-Huitrón et al., 2013; Pierozan et al., 2010). In addition to mediating excitotoxicity, QUIN acts as an oxidative stress trigger, forming highly reactive complexes by chelating free Fe^2+^, not only significantly enhancing reactive oxygen species (ROS) and hydroxyl radical generation mediated by the Fenton reaction, but also triggering lipid peroxidation chain reactions (Hestad et al., 2022; Kubicova et al., 2015). Notably, the series of oxidative stress processes described above can either be exacerbated indirectly by the NMDAR-Ca^2+^ signaling pathway or independently triggered directly by the QUIN-Fe^2+^ complex.3-HK is a precursor of QUIN and an endogenous neurotoxin that exerts neurotoxic oxidation at nanomolar concentrations to produce ROS, superoxide radicals, and hydrogen peroxide, and induces copper-dependent oxidative protein damage, leading to neuronal degeneration and programmed death (Hughes et al., 2022). In addition to this, 3-HK synergistically enhances cytotoxicity mediated by QUIN activation of NMDA receptor formation (Colín-González et al., 2014). Both 3-HK and QUIN can induce inflammation and promote the secretion of central pro-inflammatory factors, and then IDO hyperactivation promotes the accelerated rate of TRP conversion, resulting in a dramatic increase in KYN levels; while downstream KMO is also overexpressed in the inflammatory environment, thus jointly promoting the neurotoxic branch of KP imbalance, and KP falls into a vicious cycle.

In astrocytes and peripheral skeletal muscle cells, KYN is catalyzed by kynurenine aminotransferase (KAT) to generate kynurenine quinolinic acid (KYNA), and most of KYNA in the CNS is synthesized by KATII (Martin et al., 2020; Ramos-Chávez et al., 2018). In contrast to QUIN, KYNA is neuroprotective and can antagonize the neurotoxic effects of QUIN through a multi-target mechanism, and its effects form a dynamic balance with the pathological pathway of QUIN. KYNA is a non-selective NMDAR antagonist that also blocks excitatory neurotransmission of other iGluRs such as α-amino-3-hydroxy-5-methyl-4-isoxazolylpropionic acid (AMPARs), thereby counteracting QUIN-induced excitotoxicity (Mithaiwala et al., 2021). KYNA also acts as a negative allosteric modulator of α7nAChR receptors, reducing presynaptic Glu release and blocking the QUIN-induced vicious cycle of Ca^2+^ overload (Török et al., 2020; Vécsei et al., 2013). In addition to preventing QUIN-induced neuronal cytoskeletal damage (Pierozan et al., 2018), KYNA acts as an endogenous oxidant to scavenge ROS, reactive nitrogen species (Lugo-Huitrón et al., 2011), and enhances antioxidant enzyme activity to exert neuroprotective effects against QUIN neurotoxicity (Ferreira et al., 2018). However, although the neuroprotective effect of KYNA antagonizes the toxic effect of QUIN, KYNA requires up to three times the QUIN concentration to effectively neutralize its toxicity (Lovelace et al., 2016). In addition, IDO-1 in astrocytes is also overexpressed by inflammatory mediators; however, due to cell type specificity, this cell does not express KMO and cannot synthesize QUIN, which is converted to neuroprotective KYNA by highly expressed KAT; in contrast, microglia cannot synthesize KYNA due to deletion of KAT, but generate neurotoxic QUIN catalyzed by specifically expressed KMO, which presents a localized expression pattern in the CNS (Chen and Geng, 2023). In summary, KP produces KYNA or the cofactor nicotinamide adenine dinucleotide (NAD^+^) under physiological conditions, but KP shifts from QUIN/KYNA balance to over-expressing QUIN and other KP neurotoxic products under organismal inflammatory conditions (Pérez-De La Cruz et al., 2012).

## 3 KP and AD

### 3.1 KP abnormalities and AD

Studies have shown that immune dysfunction (Liu et al., 2023), Glu excitotoxicity (Wang and Reddy, 2017) and tau phosphorylation (Rawat et al., 2022) are closely related to the abnormal development of AD, while the immune system, glutamatergic system and protein kinase/phosphatase system involved in the above factors are cross-regulated through KP. KP, which is hyperactivated by pro-inflammatory factors, tends to neurotoxic branches to generate QUIN and 3-HK, among others, and plays an important role in the pathogenesis of AD through oxidative stress and/or inflammatory responses.

The academic community has long shown that KP is abnormally activated in the brain tissue of AD patients. Baran et al. reported that KYN and 3-HK contents were significantly decreased in the frontal cortex, caudate nucleus, putamen, hippocampus, and cerebellum, but significantly increased in the putamen and caudate nucleus in AD patients (Baran et al., 1999); Some scholars attribute the abnormal increase of KYNA content to the compensatory physiological response of the body and believe that it is the compensatory mechanism by which KYNA antagonizes QUIN-mediated excitotoxicity (González-Sánchez et al., 2020; Jacobs et al., 2019). Subsequently, numerous studies have shown that KP metabolites and enzymes are associated with AD. Guillemin et al. found that IDO was overexpressed and QUIN content was significantly increased in microglia, astrocytes, and neurons in the medial temporal lobe, frontal lobe, and cingulate cortex of the brain in AD patients by immunohistochemical techniques, with IDO and QUIN in microglia and astrocytes being most significantly expressed and diffusely distributed in the periphery of senile plaques; QUIN colocalized with hyperphosphorylated tau in cortical neurons and also existed evenly in NFT in the form of granular deposits (Guillemin et al., 2005) providing conclusive evidence for KP involvement in AD pathogenesis. However, the sample size of the above studies is small and needs to be further verified in combination with large-sample studies. Van der Velpen et al. used untargeted metabolomics and targeted quantification analysis to confirm that cerebrospinal fluid QUIN levels were elevated in AD patients, and microtubule-associated protein tau and threonine-181 phosphorylated tau (pTau-181) were significantly negatively correlated with cerebrospinal fluid QUIN content. B-Amyloid-42 (Aβ1-42) was significantly positively correlated with KYNA content in cerebrospinal fluid (van der Velpen et al., 2019). Bakker et al. found that plasma TRP and KYN contents were significantly negatively correlated with total tau and phosphorylated tau, cerebrospinal fluid TRP and KYN contents were significantly negatively correlated with phosphorylated tau, and cerebrospinal fluid KYN, KYNA, and KYN/TRP contents were significantly positively correlated with Aβ1-42 in AD patients using ELISA (Bakker et al., 2023). There is now evidence that Aβ1-42 induces IDO1 expression and enhances QUIN production, and significantly elevates pro-inflammatory cytokine responses, thereby upregulating IDO1, TDO2, and KMO expression (Guillemin et al., 2003; Pocivavsek et al., 2024). However, a large study reported that neuroprotective metabolites KYNA and Pic were increased in cerebrospinal fluid of AD patients and were also associated with slow progression of AD, while toxic metabolites 3-HK and QUIN were not significantly increased (Knapskog et al., 2023). The reasons for this finding may be methodological differences, or influenced by disease stage vs. population cohort characteristics. In addition, age as a factor associated with KP may also influence the study results, as controls were younger than AD patients in van der Velpen's study, which suggests that future studies need to strictly match age variables (Giil et al., 2017; Knapskog et al., 2023). It has also been demonstrated that patients with AD have decreased KYNA/QUIN ratio in cerebrospinal fluid and decreased XA/3-HK ratio in serum. Age was strongly associated with increased KYN, KYNA, and QUIN contents in serum and cerebrospinal fluid, and KYNA concentrations were significantly lower in AD patients than in age-matched controls. Serum KYN and QUIN levels are strongly positively correlated with corresponding levels in cerebrospinal fluid (Sorgdrager et al., 2019). This suggests that KP metabolite levels are altered in both the periphery and CNS of AD patients and are positively correlated with the severity of clinical symptoms, in good agreement with what has been reported in previous studies (Jacobs et al., 2019). In addition, there was a significant association between changes in peripheral levels of KP metabolites and cognitive dysfunction in AD patients. Gulaj et al. used HPLC to analyze plasma TRP and KP metabolites in AD patients and age-matched controls and confirmed that plasma TRP and KYNA levels and AA/KYN, KYNA/KYN, and 3-HK/KYN ratios were significantly decreased, and QUIN levels and KYN/TRP and QUIN/3-HK ratios were significantly increased in AD patients. Plasma TRP levels were positively correlated with instrumental activities of daily living (IADL) and physical activities of daily living scores (PADL) assessing self-care ability and independent living standards in individuals with cognitive impairment groups. AA levels were positively correlated with IADL scores. KYNA levels and KYNA/KYN ratio were positively correlated with Mini-Mental State Examination (MMSE) scores reflecting cognitive function in AD. QUIN levels were negatively correlated with clock drawing test (CDT) scores reflecting the severity of cognitive impairment in AD patients (Gulaj et al., 2010). Another study showed that age was significantly positively correlated with KYN, 3-HK, QUIN content and KYN/TRP ratio in plasma and significantly negatively correlated with XA content in AD patients. QUIN levels were significantly negatively correlated with Cambridge Cognitive Examination (CAMCOG) scores, which reflect the degree of dementia and assess the degree of cognitive impairment (Giil et al., 2017).

In summary, the levels of heavy KP metabolites in the peripheral and central nervous systems of AD change, and are closely related to the development of AD. However, research regarding KP abnormalities and aspects of AD remains problematic. (1) The results of some current studies on KP metabolite levels in AD are conflicting and lack sufficient experimental data to support them; small sample sizes in some studies and differences in detection methods between different studies may affect the generalizability of the results, (2) existing studies have not been able to determine whether KP imbalance is a causative factor or a secondary phenomenon in the development of AD. It is thought that KP imbalance may precede clinical symptoms (Knapskog et al., 2023), (3) there is also a lack of a strong explanation for the differences in KP metabolism that appear in different brain regions. In response to the above issues, further studies are still needed to elucidate the potential mechanism of action of KP in AD.

### 3.2 KP and AD treatment

The association between AD symptoms and levels of specific KP metabolites suggests that reversing KP imbalance would be beneficial in alleviating AD progression. One way to achieve this goal could be to exert a direct impact on KPs. KYNA has the limitation of low permeability through blood-brain barrier and insignificant administration effect. In animal models of AD, it has been shown that KYNA analogs or KYNA prodrugs are effective methods to prevent and/or delay AD progression. Majerova et al. used the KYNA analog kynurenoquinolinic acid N-(2-N, N-dimethylaminoethyl)−4-oxo-1 H-quinoline-2-carboxamide (KYNA-1), which has similar biological activity but is more brain permeable, to overcome BBB restriction and found that KYNA-1 reduced hyperphosphorylation of insoluble tau in the brain and plasma total tau and inhibited neuroinflammation to alleviate AD progression in SHR-24 transgenic rats when administered chronically (Majerova et al., 2022). Deora et al. developed multi-target KYNA series analogs 5b and 5c with structural modification of canine urinary quinolinic acid (KYNA) mother nucleus. In AD transgenic C. elegans strain GMC101, 5c administration significantly suppressed Aβ42 profibrotic levels and prevented Aβ42-induced cytotoxicity. 5b inhibited NMDAR and had moderate potency during DPPH radical scavenging. 5b and 5c are highly permeable in tests based on the blood-brain barrier (BBB) model of MDR1-MDCKII cells (Deora et al., 2017). It was shown that systemic administration of 4-chloro-KYN (KYNA analog), a prodrug of 7-chloro-KYNA (synthetic KYNA prodrug), protected rat hippocampus from QUIN-induced excitotoxicity (Wu et al., 2000). 3-HAA is an essential precursor for the synthesis of QUIN and has dual oxidative stress and antioxidant properties, which are associated with a variety of physiological and pathological processes. Studies have confirmed that transgenic C. elegans AD models expressing amyloid-β in body wall muscles effectively prevent Aβ toxicity after 3-HAA supplementation, and their efficacy is equivalent to direct knockdown of HAAO-1 (Hull et al., 2024).

IDO and TDO are responsible for regulating the rate of KYN production, and their activity determines the potential role of KP neurotoxic metabolites in neurodegenerative diseases, which are now confirmed as one of the key targets for AD drug therapy. Souza et al. showed that 1-MT treatment with IDO inhibitor decreased KYN content and KYN/TPR ratio in prefrontal cortex and hippocampus, and improved cognitive memory ability and non-cognitive dysfunction in Aβ1-42-induced AD model in mice. It is characterized by increased cognitive indices in novel object recognition experiments and opening and closing activity times in elevated plus maze as well as decreased immobility times in tail suspension experiments (Souza et al., 2016). Breda et al. showed that peripheral blood KYNA, 3-HK, XA, 3-HAA, and QUIN levels were lower in β-amyloid precursor protein (APP233) transgenic mice than in control wild-type mice with AD. After 6 weeks of oral administration of TDO inhibitor 680C91, peripheral blood KYN, QUIN, and PIA levels were significantly reduced, and memory recognition and spatial learning ability and anxiety-related behaviors were improved. It is characterized by a decrease in cognitive index in the novel object recognition test, a decrease in escape latency and second quadrant activity time in the Morris water maze; and an increase in open-arm activity time and a decrease in closed-arm activity time in the elevated plus maze (Breda et al., 2016). Minhas et al. showed that 4-week treatment with the IDO1 inhibitor PF068 (15 mg/kg) decreased KYN content in the hippocampus of APP/PS1, 5XFAD, and PS19 (P301S) tau transgenic mouse AD models, significantly reduced latency to reach the target hole in the Barnes maze test and significantly increased the identification index in the novel object recognition test. Aβ peptide accumulation in thioflavin S (ThioS) -positive dense core plaques and 6E10-positive diffuse plaques was significantly reduced in the hippocampus of the 5XFAD mouse model, and Thr231 phosphorylated tau in the soluble and insoluble tau fractions and Thr181 phosphorylated tau in the insoluble fraction of the hippocampus were significantly reduced in the PS19 mouse model (Minhas et al., 2024). It has also been shown that coptisine, an IDO-1 inhibitor, decreased IDO concentrations in serum and mRNA levels of IDO1, KYNU, KMO, and 3-HAAO in the hippocampus of AβPP/PS1 transgenic AD mouse models, and eliminated neuroinflammatory responses and neurotrophic defects in the hippocampus (Yu et al., 2015). KMO is a key enzyme in the toxic branch of KP and is not only directly involved in the synthesis of 3-HK, but also plays a central role in the formation and function of downstream metabolites 3-HAA and QUIN (Hughes et al., 2022). Zwilling et al. showed that KYNA levels in the brain of β-amyloid precursor protein (APP) transgenic mouse AD model were lower than those in littermate controls, and after long-term oral administration of JM6 (weak KMO inhibitor), KYNA levels in brain tissue and peripheral blood were significantly increased, and extracellular Glu in neurons in the brain was continuously reduced and synaptic loss in the hippocampus and cortex was prevented, and spatial memory deficits in the Morris water maze test and anxiety-like behavior in the elevated plus maze were improved, as shown by increased activity time in the platform quadrant and closed/open arm activity time ratio, respectively (Zwilling et al., 2011).

In summary, targeting the KP major enzyme system to regulate KP homeostasis could provide new therapeutic directions for AD. Modulators of the major KP enzymes, such as agonists or antagonists, and analogs and precursors of their major neuroprotective metabolites can be potential therapeutic targets for AD and one of the strategies to achieve effective neuroprotection in AD. However, current evidence supporting these strategies stems almost exclusively from animal model studies and no relevant human clinical studies have been identified. In addition, targeted regulation of the KP system is highly complex, and some KP metabolites play different mechanistic roles according to concentration gradient and microenvironment specificity (such as the dual characteristics of 3-HAA), so supplementation of KP metabolite analogs may trigger unpredictable pathway compensation; while long-term intervention against specific KP enzymes will likely lead to disturbance of the degradation cascade downstream of KP and affect the production of downstream products resulting in impaired KP function, and comprehensive studies are needed to evaluate the strategy of KP enzyme inhibitors and agonists and KP metabolite analogs to target KP for the treatment of AD in the future.

## 4 Exercise for AD prevention and treatment

Exercise, as a highly socially acceptable, low-cost, and low-risk non-pharmacologic intervention, has been shown to improve overall health and has a specific positive impact on brain health and is considered an effective strategy to prevent cognitive decline and reduce the risk of cognitive impairment and dementia (Brasure et al., 2018; Tao et al., 2023). Epidemiological studies have demonstrated that physical exercise is associated with a reduced risk of cognitive impairment and with behavior-related improvements in patients with neurodegenerative diseases (Marques-Aleixo et al., 2012). Lack of physical activity is generally considered a predisposing factor for AD, and overall physical activity in middle age or later life is associated with a reduced risk of AD in later life, while higher levels of exercise are associated with a lower incidence of AD (Friedland et al., 2001; Iso-Markku et al., 2022). In addition, clinical studies have confirmed that different types of exercise (aerobic exercise, resistance exercise, physical and mental exercise) can actively promote AD rehabilitation through a variety of ways, improve their physical and cognitive function in multiple dimensions, and effectively control and delay AD disease progression (Andrade-Guerrero et al., 2023; De la Rosa et al., 2020).

### 4.1 Aerobic exercise and AD exercise prevention and treatment

Among various types of exercise programs, aerobic exercise is considered the most potential and cost-effective intervention (Lee et al., 2023). Aerobic exercise such as outdoor aerobic walking (Venturelli et al., 2011), power cycling (Yu et al., 2021), and treadmill training (Vidoni et al., 2019) are widely used as adjuncts to AD drug therapy and have been shown to have direct beneficial effects in improving cognitive function, motor function, and quality of life in AD patients. Aerobic pedal rehabilitation training, based on the fixation device ReckMOTOmed, improves multidimensional cognitive function (including executive control, verbal fluency, response speed, and attention allocation) in AD patients and greatly relieves stress on care in AD patients; follow-up studies have shown that its improvement remains sustained for at least 3 months after termination of the intervention (Holthoff et al., 2015). Comparative studies have found that different aerobic exercise training modes enhance aerobic fitness and physical performance in AD patients, with intermittent aerobic training (IAT) also significantly improving self-care ability in AD patients (Enette et al., 2020). It has also been shown that even a single acute work rate cycling training session significantly improves cognitive dysfunction in patients with moderate AD, and this improvement is enhanced when combined with cognitive play (Ben Ayed et al., 2021). Subsequent studies have shown that there is also no significant gender difference in the benefit of a single acute aerobic exercise in AD patients (Ben Ayed et al., 2024) (as shown in Table 1).

**Table 1:** Research on the relationship between aerobic exercise and AD prevention and treatment.

**Authors**	**Sample size**	**Age**	**Experimental group**	**Control group**	**Main result**
(Venturelli et al. 2011)	Walking program (11) Control (10))	83 ± 6	Aerobic walking: Walking with hands outside accompanied by nursing staff. 30 min/time, once a day, 4 times a week, 6 months.	Control group: Bingo games, spelling sewing and music therapy were performed every day. 30 min/time, once a day, 6 months.	Six-minute walk test (6 MW) total distance and activities of daily living (ADLs) activities of daily living index (BI) were significantly increased.
(Yu et al. 2021)	Power cycling group (64) Stretching control group (32)	66–85	Power cycling: intensity of 50–75% heart rate reserve (HRR) or subjective fatigue perception assessment scale (Borg RPE) 9–15. The initial phase starts with 50–55% HRR or RPE9-11and increases by 5 min or 5% HRR weekly until the target of 70–75% HRR (RPE12–14) and 50 min is reached. 30–50min/time, warm-up and relaxation for 5 min each,4 times/week, lasting for 6 months.	Control group: Sitting exercise and static stretching exercise were performed. Less intense than HRR < 20% or Borg RPE 9. The number of repetitions and duration of each stretch gradually increased and the duration was the same as in the power cycling group.	Alzheimer's Disease Assessment Scale-Cognitive Subscale (ADAS-Cog) scores were significantly reduced.
(Vidoni et al. 2019)	Treadmill group (33) Stretch conditioning control group (32)	>55	Treadmill: 40–55% HRR in target heart rate interval (THR) for first 4 weeks; 50–65% HRR for weeks 5–18; 60–75% HRR for last 8 weeks. Incremental slope and speed warm-up for 5 minutes, decreasing slope and speed for 5 minutes at the end. Exercise was performed 60 min per week from week 1 and increased approximately 21 min per week to reach a target of 150 min per week after 3–5 training sessions. 30–50 min/time, 60–150 min/week for 26 weeks.	Control group: A series of weekly rotations of non-aerobic exercise (core intensive training, elastic belt, modified Tai Chi, modified yoga) were performed after a 5-min hot walk on the runway. Participants wore a heart rate monitor and required a THR of less than 100 bpm.	Dementia Disability Assessment Scale (DAD) and IADL scores were significantly elevated.
(Holthoff et al. 2015)	PA Intervention (13) Control (14)	≧55	PA Intervention: ReckMOTOmed lower limb rehabilitation training machine was used. Exercise trainer resistance level set to 2–4. 30 min/time, 1 time/day, 3 times/week for 12 weeks.	Control group: received only usual care.	Semantic and phonemic word fluency measured in Alzheimer's Disease Registry Consortium Test (CERAD) and Letter Fluency Test (FAS) increased significantly, and ruler dropping test predicted significantly less time; ADL total score and NPI total score remained stable during the study and follow-up periods.
(Enette et al. 2020)	CAT (14) IAT (17) Control (21)	68–82	CAT: Cycling was performed with intensity set according to incremental maximal exercise test (IMET) results (70% HRmax for achievers and 50% MTP for non-achievers) and dynamic incremental loading of 5–10 W according to heart rate reduction of 5–10 beats per minute or RPE reduction of 1 grade (Borg 6–20). IAT: 6 × 1min high-intensity cycling exercise (PEAK: 80% HRmax or MTP-10W) + 4 min recovery (BASE: 60% HRmax), increasing 5–10W according to heart rate decreased 5–10 beats/min or RPE decreased 1 grade (Borg 6–20), and heart rate at 28th/30 min was used as the regulation benchmark throughout the training. 30 min/time, twice a week for 9 weeks.	Control group: not participating in any exercise training program. Weekly meetings and questionnaire activities were held for a total of 9 meetings of 30 min each.	After CAT and IAT interventions, 6 MW walking distance and IMET maximum task metabolic equivalent (MET) increased; CAT also increased Alzheimer's Disease Quality of Life Scale (QoL-AD) score.
(Ben Ayed et al. 2021)	EG (27) CEG (26) CG (26)	69.62 ± 0.99	EG: Performed a 20-min ergometer bicycle exercise at an intensity equivalent to 60% of the maximum heart rate at the end of the 6 MWT test. Warm up for 5 min and relax for 5–10 min. CEG: Performing a 20-min power cycling exercise while completing a simple computer cognitive game. Warm up for 5 min and relax for 5–10 min.	CG group: perform 20 min reading task	The Stroop test interference score and the completion time of Hanoi tower task were significantly reduced, and the correct rate of memory span forward and backward test was significantly increased.

In conclusion, aerobic exercise, as a mainstream intervention for cognitive rehabilitation, shows immediate benefits in symptom management in AD patients and is regarded as one of the potential non-pharmacological intervention strategies. However, large-scale randomized controlled trials with long-term follow-up are needed to confirm the current findings, and the optimal intensity-frequency combinations and optimal implementation protocols for different training modalities (e.g., continuous training vs. intermittent training) remain to be further explored and evaluated.

### 4.2 Resistance training and AD exercise prevention and treatment

Resistance training is a periodic form of physical exercise that induces neuromuscular adaptive changes by gradually overload of skeletal muscle using external weights and is considered an important intervention to improve most motor performance (Sepúlveda-Lara et al., 2024). It has been suggested that diminished muscle strength may be an early indicator or contributory factor in the development of Alzheimer's disease (Buchman et al., 2007). Lower muscle strength is associated with an increased risk of cognitive impairment, including AD (Boyle et al., 2009). Whereas, for every unit increase in muscle strength, the prevalence of AD decreases by 43% at the beginning of cognitive impairment (Li et al., 2024).

Resistance training has been shown to positively impact agility, lower limb strength, balance, and flexibility in AD (Garuffi et al., 2013). Papatsimpas et al. demonstrated that resistance training was effective in slowing global cognitive function, executive function, and working memory decline and improving instrumental daily activity in patients with mild AD; the effect was more significant if combined with aerobic exercise (Papatsimpas et al., 2023). Xiao et al. showed that isokinetic muscle strength training can not only significantly promote the recovery of cognitive function and activities of daily living in AD patients, but also significantly improve motor function and balance function, and advocated that isokinetic muscle strength training should be used as an adjuvant therapy for the treatment of AD patients (Xiao et al., 2017). Chang et al. showed that resistance training targeting the trunk and lower limbs resulted in significant improvements in depression, muscle mass, and muscle function in patients with mild AD-sarcopenia comorbidity (Chang et al., 2020). Resistance training of the upper and lower extremities based on elastic bands has also been shown to significantly improve muscle strength and endurance, cardiopulmonary function, and gait speed in patients with mild AD, and resistance training is considered an effective rehabilitation program for patients with AD (Ahn and Kim, 2015). Other studies have also shown that resistance training does not significantly improve cognitive function in AD patients. The reason for the lack of expected results may be that the low-volume, low-intensity exercise program adopted under the principle of safety first failed to fully activate the neural adaptive mechanism (Vital et al., 2012). Based on the multifaceted improvement of AD patients by resistance training with different intensity and cycle parameters mentioned above (including cognitive function, motor function, muscle strength, functional flexibility, and quality of life), it should be considered as an important component of AD public health promotion programs (as shown in Table 2).

**Table 2:** Research on the relationship between Resistance training and AD prevention and treatment.

**Authors**	**Sample size**	**Age**	**Experiment group**	**Control group**	**Main result**
(Garuffi et al. 2013)	Resistance training (TG) group (17) Social gathering group (SGG) (17)	78.2 ± 7.3	TG: Training consisted of 5 device exercises (chest clipper, high pulldown, leg lift, triceps pulley press, and barbell bending) for major muscle groups; initial load was determined by two sets of 20 + exhaustive tests (more than 22 with dosing increases and retesting every 15 days), with an adaptation period for the first 5 weeks. Each training session was warmed up with low load and completed 3 sets × 20 times with 85% maximal load (2 min rest between groups) without stretching throughout. 60 min/time, 3 times/week, non-continuously for 16 weeks.	SGG: Conducted in a quiet environment, activities include painting, writing, reading, group activities and relaxation training, and occasionally walking (non-regular) in track and field. 60 min/time, 3 times/week, non-continuously for 16 weeks.	Basic activities of daily living (Basic ADLs, BADLs) and IDAL scores significantly increased.
(Papatsimpas et al. 2023)	Aerobic and Resistance Exercise (57) Resistance Exercise (57) Control (57)	≧65	Aerobic exercise and resistance exercise: perform moderate intensity step (64–76% HRmax, 30 min/time, 5 times/week, for 12 weeks), with resistance training, the movement covers the main muscle groups such as biceps bending, shoulder flexion and extension, hip and knee extension (50–69% 1-RM, 10 movements × 2 groups × 12 times, interval between groups 1–3 min, 30–45 times/min, 5 times/week, for 12 weeks, including 1 home, 2 supervision in the rehabilitation center). Resistance exercise: Performed the same resistance training as the upper group.	Keep your daily activities away from any exercise program.	The Andenbrook Cognitive Examination (ACE-R), Digit Span Forward Backward Test (DST F-B), and IDAL scores significantly increased, and the Trail Making Test A-B (TMT A-B) took significantly less time.
(Xiao et al. 2017)	Conventional therapy combined with isokinetic muscle strength training group (20) Conventional therapy group (20)	69–78	On the basis of routine clinical treatment (the same as routine treatment group), isokinetic muscle strength rehabilitation training was performed. Isokinetic muscle strength training was performed on the quadriceps femoris and hamstrings of the lower limbs using the Kinitech isokinetic system using an alternate day training method. According to the patient's condition, select 60-150°/s angular velocity (such as 60/90/120 or 90/120/150 combination), repeat each group for 10 times, rest for 20 seconds between groups, rest for 2 minutes between cycles, and complete 2–4 cycles daily (it is appropriate to induce moderate fatigue on the day and no fatigue on the next day). Each muscle group was trained once a day, 3 times/week for 8 weeks.	Routine treatment was oral donepezil hydrochloride 5 mg/day, and symptomatic treatment such as antiplatelet, lipid-lowering, antihypertensive, or hypoglycemic therapy was given as appropriate.	CAMCOG, Berg balance test scores, and functional extension test distance significantly increased, and timed up-and-go test (TUG) time significantly decreased.
(Chang et al. 2020)	Exercise (20) Control (20)	79 ± 5.1	Resistance training: Including 10-min warm-up, 40-min Theraband elastic band resistance exercise (intensity set at the patient's self-perceived “moderate exertion” level) and relaxation links, focusing on strengthening the trunk and limb core muscle groups. 50 min/time, 3 times/week, non-continuous day training for 12 weeks. Control group: no training.	Control group: no training.	Hamilton Depression Rating Scale (HDRS) and Beck Depression Inventory (BDI) scores were significantly reduced, and isometric maximum voluntary contraction (N/kg), grip strength, and gait speed were significantly increased in shoulder abduction, hip and elbow flexion, and knee extension.
(Ahn and Kim 2015)	Experimental group (23)	74.21 ± 6.09	Resistance training: consisted of 10 min warm-up, 10 min relaxation, and 40 min progressive upper and lower limb resistance exercises based on Theraband elastic bands. Upper limb training included seven movements: sitting rowing, overhead pushing, biceps bending, shoulder forward flexion 90 degrees, PNF D2 flexion mode, elbow flexion training, and back shoulder archery pulling, with 10 times × 3 groups of lower limb training including seven movements: hip flexion and extension, heel lift training, device leg lifting, hip adduction in standing knee extension, hip abduction with external rotation in standing position, and ankle plantar flexion in long sitting position, using the same 10 times × 3 group mode. 60 min/time, once a day, 3 times a week for 5 months.	Pre-and post-control within group	The number of chair squats, left and right one-legged standing time (UST), total steps in the 2-min walking test, and gait speed in the 8-m walking test were significantly increased.

### 4.3 Physical and mental exercise for AD prevention and treatment

Physical and mental exercise is a non-pharmacological adjunctive strategy to improve symptoms in AD patients. Traditional Chinese physical and mental exercises such as Tai Chi and Baduanjin Qigong are mostly based on Traditional Chinese Medicine (TCM) theory, combined with respiratory control, stretching and relaxation of skeletal muscles and concentration of mental ideation, which aim to enhance strength, flexibility, balance and proprioception, while increasing attention to reduce anxiety and stress, and have a positive effect on improving cognitive impairment, physical function and quality of life in AD patients (Jiang et al., 2022). Yao et al. found that Tai Chi significantly improved functional activity performance associated with fall risk and effectively reduced fall risk in AD patients (Yao et al., 2013). Li et al. showed that Tai Chi exercise combined with Naoling decoction improved cognitive function and anxiety status and quality of life in AD patients (Liu et al., 2013). It has also been confirmed that Tai Chi combined with traditional Chinese art calligraphy and painting exercises that have a stabilizing physical and mental effect can significantly improve cognitive function and quality of life in AD patients (Tai et al., 2016). In addition, Baduanjin Qigong has been shown to positively improve cognitive function and activities of daily living in patients with mild-to-moderate AD (Wei et al., 2024). In recent years, dance and yoga have also been used in the rehabilitation of AD patients and have been shown to show positive benefits in improving cognitive function, neuromental status, balance ability, functional flexibility, and lower limb strength in AD patients (Tao et al., 2023). Chiesi et al. showed that Biodanza intervention significantly improved neuropsychiatric symptoms in AD patients and relieved aggressive behavior and verbal agitation symptoms in agitated states, with a positive promoting effect on mental health status (Chiesi et al., 2021). Salsa dance has also demonstrated significant improvements in range of motion, strength, balance, functional flexibility, gait distance, and speed in AD (Abreu and Hartley, 2013). In addition, yoga significantly improves cognitive function and quality of life in AD patients, while effectively relieving depressive symptoms and enhancing their social engagement. This therapy not only has a positive impact on mental health and overall quality of life in AD patients, but also significantly reduces caregiver stress (Kaushik et al., 2025) (as shown in Table 3).

**Table 3:** Research on the relationship between physical and mental exercise and AD prevention and treatment.

**Authors**	**Sample size**	**Age**	**Experiment group**	**Control group**	**Main result**
(Yao et al. 2013)	Tai Chi (22)	80.6 ± 6.2	Tai Chi: In the first stage, simplified Yang Tai Chi course training was performed, 60 min/time, twice a week, with an interval of 1 to 3 working days between each class, for 2 weeks; in the second stage, home Tai Chi exercise was performed with the assistance of nursing staff, 20 min/time, three times a week, for 12 weeks.	Pre-and post-control within group	TUG and UST time increased
(Liu et al. 2013)	Naoling decoction combined with Tai Chi (32) conventional treatment group (30)	52.5 ± 10.8	The treatment group was additionally treated with Naoling Decoction on the basis of conventional treatment; 1 dose per day, decoction, divided into 2 doses, if necessary, can be added or subtracted with the disease; at the same time, Yang's simplified Tai Chi 24-style exercise, 5 times/week, for 3 months.	The control group was treated with conventional methods, oral piracetam tablets, 400 mg, 3 times/day; oral nimodipine, the initial dose of 20 mg, 3 times/day, 3 days later without adverse reactions gradually increased to 40 mg, 3 times/day, and conventional rehabilitation training such as memory thinking training and self-care ability training.	MMSE and ADL scores were significantly increased, and Hamilton Anxiety Rating Scale (HAM-A) scores were significantly decreased.
(Tai et al. 2016)	Intervention (14) Control (10)	>65	24 style Yang Tai Chi 1 h, calligraphy 1 h, painting 1 h. 3 h/time, twice a week for 6 weeks.	Maintain daily activities. Participants in the control group performed non-health-related social activities such as playing cards and singing during the same time period.	Increased scores on the Cognitive Performance Screening Instrument (ACASI) Orientation Subscale and the World Health Organization Quality of Life-BREF (WHOQOL-BREF) Psychiatry Sub item.
(Wei et al. 2024)	Baduanjin (48) control group (50)	73.09 ± 7.18	Baduanjin: A structured Baduanjin intervention was implemented on the basis of the control group, including a complete 10 style such as preparatory type and two hands supporting Tianli Triple Jiao, which was performed during 14:30-−18:30 every day, 30 min/time, 7 times/week, for 4 weeks, and patients were provided with Baduanjin teaching videos at discharge, and family members were followed up by WeChat/telephone to supervise home training to ensure the continuity of the intervention.	Control group: 4 weeks of routine care, including diet care, safety protection, sleep care, psychological care.	MMSE, AD Collaborative Study Daily Performance Scale(ADCS-ADL)and QOL-AD were significantly increased.
(Chiesi et al. 2021)	Biodanza (16)	80.2 ± 7.5	Biodanza: It is divided into two stages, the first stage (2 h/time, once a week for 3 months) and the second stage (1 h/time, once a week) courses are performed with the assistance of specialists. Appropriate music types and movements were also selected according to the patient's physical and mental status.	Pre-and post-control within group	Neuropsychiatric Inventory—Nursing Home Version (NPI-NH) and Cohen-Mansfield Agitation Inventory (CMAI) scores for aggression and verbal agitation were significantly reduced.
(Kaushik et al. 2025)	Yoga (30)	66.4 ± 3.8	Yoga: 60 min/session, 6 sessions/week for 12 weeks. Including 10 min of bay Japanese, 5–7 min of deep muscle relaxation, 15 min of asana (supine, prone, sitting, and standing positions), 15 min of pranayama, and the last 5–10 min of meditation ended.	Pre-and post-control within group	GDS scores decreased significantly and MoCA overall scores increased significantly.

In summary, various types of exercise therapy have potential clinical benefits in the overall management of AD. Although there is no consensus on improving the optimal type and intensity of exercise in AD patients to date, it has been comprehensively documented that regular moderate-intensity exercise or combined exercise (e.g., aerobic exercise plus resistance training), and maintaining a high frequency (e.g., >30 min per day, ≥2 days per week for ≥4 weeks), can bring significant clinical benefits to delay the progression of AD. Key implementation principles include the development and implementation of personalized exercise prescriptions under the guidance of professionals, whose content, intensity, and supervision level need to be adjusted in a timely manner according to individual health status and needs, especially considering the staging characteristics of AD, and early patients are recommended to pursue the benefits of maximizing cognitive and physiological function improvement and delaying decline using moderate-intensity exercise (e.g., brisk walking, power cycling) or combined training (e.g., aerobic resistance + resistance) under the premise of assessing good tolerability. For patients in the middle and advanced stages, in view of their significant cognitive and motor dysfunction, it is necessary to give priority to ensuring safety and compliance. Physical and mental movements (such as yoga, tai chi, qigong) emphasize physical and mental integration, and have low fall risk characteristics and adaptability to residual executive function, which can be used as a core intervention for the management of behavioral psychiatric symptoms (BPSD) and maintenance of ADL in patients with advanced AD. Exercise protocols must also be developed to integrate patients' current physical condition exercise type preferences, and previous medication use to ensure compliance and long-term sustainability. Although the current AD exercise intervention strategy has certain applicability, there are few randomized controlled studies on the effect of different types of exercise on improving the symptoms of AD patients, and it is necessary to compare the improvement effect of different types of exercise on the symptoms of AD patients through a larger sample size. This requires that future studies should focus on the following: (1) consider combined exercise and focus on optimizing the dose, and investigate whether specific characteristics of AD patients are associated with different types of exercise; (2) clinical intervention programs for AD patients need to fully consider specific factors such as gender, disease severity, specific drug use, and intervention cycles to effectively control heterogeneous factors and make clinical exercise intervention programs objective, scientific, and effective for AD patients; (3) evidence-based recommendations on which exercise is most suitable for an individual and the optimal length and intensity of intervention required to produce clinically relevant positive effects remain lacking, and it is necessary to systematically determine the most effective treatment for specific signs and symptoms from all available exercise types and provide individual evidence-based recommendations for AD patients.

## 5 Exercise and KP

A large number of studies have reported that exercise activates peripheral KP, and both acute (single) exercise and long-term (multi-week structured training) exercise significantly alter peripheral KP-related metabolite levels. Acute exercise such as full marathon (Lewis et al., 2010) resulted in a significant decrease in plasma TRP and a significant increase in KYN; plasma KYN was effectively converted to KYNA after cross-country running (Puigarnau et al., 2022), super marathon (Mieszkowski et al., 2022), and 150 km bicycle timer (Schlittler et al., 2016), as shown by an increase in KYNA/KYN ratio. Chronic exercise, on the other hand, similarly activates peripheral KPs, thereby increasing the propensity of KP metabolites to KYNA branching, possibly due to elevated expression levels and activity of KATs. Chronic exercise such as 72 weeks of diving training and swimming training decreased TRP content and increased KYNA content in peripheral blood (Sánchez Chapul et al., 2022). Studies have shown that the results of acute and chronic exercise on peripheral KP metabolites in clinically ill people are consistent with those in healthy people. Mudry et al. showed that a significant increase in serum KYNA concentration, a significant decrease in KYN concentration, and a significant decrease in serum TRP concentration at 3 h after recovery were observed in male patients with type 2 diabetes after performing acute aerobic exercise and a significant increase in the [KYNA] 1,000/[KYN] ratio (Mudry et al., 2016). Herrstedt et al. showed that 12 weeks of aerobic exercise and systemic resistance exercise decreased serum 3-HK concentrations in patients with gastroesophageal junction adenocarcinoma; a trend toward increased serum KYNA concentrations was found after 60 min of a single exercise (Herrstedt et al., 2019). However, not all studies have shown positive benefits of exercise for KP. Hennings et al. showed that 1 week of physical activity only significantly reduced KYN content in patients with major depression, while KP metabolites did not change significantly in the serum of patients with somatoform syndrome (Hennings et al., 2013). The emergence of such differential results suggests that the effect of exercise on KP may depend on the mode, intensity, duration of exercise, and the specific disease context of the subject, and future studies are needed to further dissect these variables. The effects of exercise on peripheral KP-related metabolic enzyme activity and expression levels have been reported in healthy organisms and different disease states. Wyckelsma et al. showed that 3-week sprint interval training increased KATIII expression levels in skeletal muscle by about 50% in healthy older men compared with controls (Wyckelsma et al., 2021). Allison et al. showed that 12 weeks of combined (systemic resistance + high-intensity interval training) exercise (significantly up-regulated KAT I–KAT IV expression levels in skeletal muscle of healthy elderly men (Allison et al., 2019). Pal et al. showed that 72 weeks of device-based progressive resistance could indirectly decrease the KYN/TRP ratio, an indicator of IDO/TDO enzyme activity, in peripheral serum of pancreatic cancer patients (Pal et al., 2021) (as shown in Table 4). These findings together suggest that different types of long-term exercise can significantly up-regulate KATs expression levels in skeletal muscle of healthy people or decrease IDO/TDO activity indicators in the periphery of diseased people, skewing KP-prone neuroprotective branches.

**Table 4:** The effect of exercise on peripheral KP enzymes and metabolites.

**Authors**	**Sample size**	**Exercise training**	**Main result**			**Tissue type**
			**Within analysis**	**Within analysis**	**Between analysis**	
(Lewis et al. 2010)	Full Marathon (25)	Marathon: 26.2 miles, average time 247 ± 46 min.	Marathon: TRP↓ KYN↑ QUIN↑ AA↑	/	/	Plasma
(Puigarnau et al. 2022)	Trail running(33)	Trail running: 21 kilometers in total, with a cumulative uphill gradient of 1,400 m.	trail running: KYN↑ KYNA↑	/	/	Plasma
(Mieszkowski et al. 2022)	Vitamin D supplementation + ultra-marathon (S) (16) Placebo + ultra-marathon (C) (19)	S: The supplement group took a single dose of vitamin D3 (150,000 IU) 24 hours before the ultra-marathon race. C: The control group took a placebo before the ultra-marathon race.	S: TRP↓ KYN↑ 3-HK↑ KYNA↔ QUIN↔ PA↔ XA↔ KYNA/KYN↔	C: TRP↓ KYN↑ KYNA↑ QUIN↑ 3-HK↑ PA↑ XA↑ KYNA/KYN↑	S vs. C: TRP↓ KYNA↓ XA↓ QA↓ PA↓ KYNA/KYN↓	Plasma
(Schlittler et al. 2016)	150 km cycling(9) Half marathon (11)	Road cycling: Amateur athletes complete the race (distance: 150 km) Half marathon: Mountain route (distance not specified)	150 km cycling: KYNA↑ QUIN↑ QUIN/KYNA↓	half-marathon: KYNA↑	150km cycling vs. half-marathon: KAT1 mRNA↑ KAT2 mRNA↑ KAT3 mRNA↑ KAT4 mRNA↑ KAT1↑ KAT3↑ KAT4↑	Plasma Muscle
(Sánchez Chapul et al. 2022)	Diving group (Divers) (20) Swimming rescue group (SHRS) (14) Control group (Controls) (12)	Divers: Underwater training: 3 pool dives per week (1–5 m deep, 2 h per session) + 3–4 deep-sea dives per week (60 m deep, 1.5 h per session), using compressed air (21% O_2_, 79% N_2_). Land Training (same as swimming group): 2 sessions per week, 20 min per session, heart rate intensity 60–80% of maximum, including strength training (push-ups, squats, etc.) and endurance training (sprinting, dragging weights). SHRS: Water training: Timed swimming (800 meters in 14 min, 1,500 m in 28 min, 2000 m in 42 min with equipment) + body-dragging training (800 m in 28 min) + 3 sessions per week of 25-m breath-holding training. Land training: Same as the diver group. Controls: Sedentary healthy males not participating in any exercise program, serving as a baseline reference.	Divers: 3-HK↑	SHRS: TRP↓ 3-HK/TRP↑	Divers vs. Controls: TRP↓ 3-HK↓ SHRS vs. Controls: KYN↓ KYNA↑ HK↑ KYNA/TRP↑ 3-HK/TRP↑	Plasma
(Mudry et al. 2016)	Type 2 diabetes group (T2D) + power cycling exercise (27) Normal glucose tolerance group (NGT) (26)	T2D: Participate in acute power cycling exercise. Warm-up: 5 min, initial load set at 50% of power output when respiratory exchange ratio (RER) = 1.0 during individual maximal oxygen uptake test. Main exercise: 30 min of continuous cycling, load adjusted to maintain 85% of maximal heart rate. NGT: Same as T2D	T2D: TRP↓ KYN↓ KYNA↑ [KYNA] ^*^ 1000/[KYN]↑	NGT: TRP↓ KYN↓ KYNA↑ [KYNA] ^*^ 1,000/[KYN]↑	T2D vs. NGT:s TRP↔ KYN↔ KYNA↔	Plasma
(Herrstedt et al. 2019)	Patients with adenocarcinoma of the gastroesophageal junction (GEJ) + aerobic interval training and resistance training (EX) (18) GEJ cancer patients (CON) (25)	EX: Aerobic interval training (stationary bike) + resistance training (chest press, leg press, lateral pull, knee extension). Frequency: 2 times per week for 12 weeks. Single session duration: 30–45 min of aerobic exercise + resistance training (total duration approximately 60 min). Intensity: Aerobic: Load set based on initial Wattmax test (high-intensity interval). Resistance: Load set based on 1RM test (4 exercises, major muscle groups). Advanced: Adjust load after mid-term assessment (ensure progressive overload).	GEJ+EX: TRP↓ AA↑	CON: TRP↓ AA↑ 3-HK↑ QUIN↑ 3-HK/KYN↑	GEJ+EX vs. CON: TRP↔ KYN↔ KYNA↔ 3-HK↓ QUIN↓ HK/KYN↓ KMO↓	Plasma Muscle
		CON: Standard care: Routine follow-up, allow participation in community exercise (no structured intervention).			
(Hennings et al. 2013)	Major depressive disorder (MDD) + EX (38) Somatoform disorder (SSI-8) + EX (27) Healthy controls + EX (58)	EX: Active Week; Home-based independent training. Perform moderate-intensity endurance training (such as brisk walking or jogging) and stretching exercises (focusing on the back, abdomen, and thigh muscle groups). 30 min per day, for 7 consecutive days.	MDD+EX: TRP↔ KYN↔ 5-hydroxyindole acetic acid(5-HIAA)↔	SSI-8+EX: TRP↔ KYN↔ 5-HIAA↔	MDD+EX vs. SSI-8+EX TRP↔ KYN↔ 5-HIAA↔	Plasma
(Wyckelsma et al. 2021)	Antioxidant group (Vitamin-treated) + EX (11) Placebo group (Placebo) + EX (9)	Vitamin-treated: Daily supplementation with vitamin C (1 g) + vitamin E (235 mg) EX: Sprint interval training. Intensity: Full-power cycling. Single training structure: After warm-up, 4–6 sets × 30 s of full-power cycling (Wingate test), with 4 min of rest between sets, 3 times a week for 3 weeks (9 training sessions in total). Placebo: Placebo administration.	Vitamin-treated+EX: TRP↔ KYN↔ KYNA↔ QUIN↔ 3-HK↔ PIC↔ KYN/TRP↔ KYNA/QUIN↔ KAT III↔s KAT I↔ KAT IV↔ TDO2↔	Placebo+EX: TRP↔ KYN↔ KYNA↔ QUIN↓ 3-HK↔ PIC↔ KYN/TRP↔ KYNA/QUIN↑ KAT III↑ KAT I↔ KAT IV↔ TDO2↔	Vitamin-treated+EX vs. Placebo+EX: QUIN↑ KYNA/QUIN↓ KAT III↓	Plasma Muscle
(Allison et al. 2019)	Healthy, non-depressed elderly men + EX(25)	EX: Perform resistance training and high-intensity interval training (HIIT) 3 times per week (2 resistance training sessions + 1 HIIT session), for 12 weeks. Resistance training: 2 times per week, warm-up (5 min of cycling followed by 3 sets of 4 exercises (leg press, bench press, etc.), intensity: 65%−80% of 1RM. HIIT: 1 time per week, warm-up for 3 min followed by 10 sets of 60 s (90% of maximum heart rate, ≥90 rpm), with 5 min of cool-down between sets.	Healthy, non-depressed elderly men + EX: KYN↓ KYNA↑ QUIN↓ QUIN/KYNA↓ KAT1↑ KAT2↑ KAT3↑ KAT4↑	/	/	Plasma Muscle
(Pal et al. 2021)	Supervised training group (7) Home-based training group (14) Control group (11)	Supervised: Resistance training with equipment, moderate to high intensity (60–80% 1-RM), twice a week, supervised by a therapist, for 6 months. Home-based: Home-based bodyweight/resistance band training (Borg scale 14–16 points, moderate intensity), twice a week, for 6 months.	Supervised: KYN↔ TRP↔ KYN/TRP↓	Home-based: KYN↑ TRP↔ KYN/TRP↑ Control: KYN↔ TRP↔ KYN/TRP↔	Supervised vs. Control: KYN↔ TRP↔ KYN/TRP↔ Home-based vs. Control: KYN↑ TRP↔ KYN/TRP↑	Plasma

↑ Significant increase (p ≤ 0.05).

↓ Significant decrease (p ≥ 0.05).

↔ No significant change.

/ No relevant data.

Reports on the effects of exercise on KP in the central nervous system have focused on rodent models. Souza et al. showed that 8-week swimming training effectively prevented cognitive and non-cognitive dysfunction and significantly decreased IDO activity, KYN and TRP content, and KYN/TRP ratio in prefrontal cortex and hippocampus of AD model mice (Souza et al., 2017). Liu et al. demonstrated that 4 weeks of swimming training improved depression-like behavior induced by chronic unpredictable mild stress (CUMS) in rats and significantly reduced IDO activity in prefrontal cortex (Liu et al., 2013). Liu Ruilian et al. found that 8 weeks of moderate-intensity treadmill exercise significantly improved neurocognitive impairment and significantly decreased IDO activity and KYN content in the hippocampus of CUMS mice (Liu and Qu, 2021). Ieraci et al. showed that 4 weeks of spontaneous running wheel exercise significantly increased KYNA content and significantly up-regulated KAT2 and KAT4 mRNA and protein expression levels in the hippocampus of homozygous knock-in brain-derived neurotrophic factor Val66Met (BDNF Met/Met) mice (Ieraci et al., 2020). Agudelo et al. demonstrated that skeletal muscle-specific peroxisome proliferator-activated receptor gamma coactivator 1α (PGC-1α) transgenic mice were significantly resistant to depression-like behavior induced by chronic mild stress (CMS) compared with wild-type mice, with significantly reduced 3-HK expression levels in whole brain tissue and significantly down-regulated IDO1, TDO1, KMO, 3-HAO, and KYNUmRNA expression levels in the hippocampus. However, skeletal muscle-specific PGC-1α gene-null mice were sensitive to CMS-induced depression-like behavior, and hippocampal IDO1, TDO1, KMO, 3-HAO, and KYNU mRNA expression levels were significantly up-regulated. However, 8-week locomotor running wheel exercise significantly upregulated PGC-1α expression in skeletal muscle of wild-type mice, suggesting that exercise can regulate peripheral and central KP metabolism by up-regulating PGC-1α expression in skeletal muscle (Agudelo et al., 2014). Human studies have found that 4 weeks of high-intensity exercise significantly increased KYNA and PIC contents, significantly increased KYNA/TRP ratio, and significantly decreased 3-HK and QUIN contents in the cerebrospinal fluid of healthy male college students; while low-moderate intensity exercise also significantly increased KYNA content, significantly decreased 3-HK and QUIN contents, and significantly decreased KYN/TRP ratio in the cerebrospinal fluid of healthy male college students (Isung et al., 2021) (as shown in Table 5). In summary, exercise with different exercise intensity, frequency and duration can change peripheral and central KP-related metabolites and enzymes. However, the current study still faces the following limitations: (1) the differential effects and long-term effects of different exercise modalities (types, intensities, etc.) on the pharmacokinetics of peripheral and central KP still need to be systematically explored; (2) the mechanism of action of exercise on central KP mainly focuses on animal models, while the limited human data (such as cerebrospinal fluid studies) have a small sample size, and whether cerebrospinal fluid parameters can accurately reflect the metabolic status of KP in the brain still needs to be verified. Future studies are needed to further explore the long-term specific effects of different exercise modalities on central and peripheral KP metabolic dynamics by combining non-invasive neuroimaging and metabolomics techniques in a well-characterized large-scale population to elucidate the possible pathways of kinesia-regulated KP (e.g., muscle-brain axis or entero-brain axis) and their mediated neuroprotective mechanisms.

**Table 5:** The effect of exercise on KP enzymes and metabolites in the central nervous system.

**Authors**	**Sample size**	**Exercise training**	**Main result**			**Tissue type**
			**Within analysis**	**Within analysis**	**Between analysis**	
(Souza et al. 2017)	Aβ sedentary group (8) Sham surgery sedentary group (8) Aβ exercise group (8) Sham surgery exercise group (8)	Swimming training: 5 times/week for 8 weeks. Week 1: Adaptation period with a water depth of 5 cm, no weight, 20 min/day. Weeks 2-3: Water depth of 20 cm, mice unable to touch the bottom, no weight, 30–40 min/day. Weeks 4–8: Progressive tail weight (1%−3% of body weight), 50–60 min/day.	Aβ sedentary vs. Sham sedentary: TRP↑ KYN↑ KYN/TRP↑ IDO↑ TRP↑ KYN↑ KYN/TRP↑ IDO↑	Aβ exercise vs. Sham exercise: KYN↑ KYN/TRP↑ IDO↑	Sham exercise vs. sham sedentary lifestyle: KYN↓ KYN/TRP↓ IDO↓ Aβ exercise vs. Aβ sedentary lifestyle: KYN↓ KYN/TRP↓ IDO↓	Prefrontal Cortex Hippocampus
(Liu et al. 2013)	Control group (CON) (10) CUMS group (10) Swimming control group (Con+Swim) (10) Swimming CUMS group (CUMS+Swim) (10)	Weightless free swimming: Adaptive training prior to formal experimentation; Week 1: Gradually increase from 15 min per day to 60 min per day. Formal training: 60 min per day, 9:00–11:00 a.m. daily, 5 days per week, for 4 weeks.	Con+Swim vs. Con: IDO↓	CUMS+Swim vs. CUMS: IDO↓	CUMS vs. Con: IDO↑ CUMS+Swim vs. Con+Swim: IDO↔	Prefrontal Cortex
(Liu and Qu 2021)	Blank control (CG) (14) Model control group (chronic stress modeling (CUMS) + no exercise) (MG) (14) Model exercise group (CUMS + exercise) (ME) (14)	Treadmill exercise: Adaptive training on a treadmill was conducted prior to the formal experiment. Moderate intensity (0% incline, speed 10 m/min) was used, 6 days/week, 60 min/day, for 8 weeks.	MG vs. CG: KYN↑ IDO↑ IDO mRNA↑	/	ME vs. MG: KYN↓ IDO↔ IDO mRNA↓	Hippocampus
(Ieraci et al. 2020)	BDNFVal/Val+sedentary (VV-SED) (17–23) BDNFVal/Val+exercise (VV-EXE) (17–23) BDNFMet/Met+sedentary (MM-SED) (17–23) BDNFMet/Met +exercise(MM-EXE) (17–23)	Voluntary wheel running: 24 h of free wheel running per day for 4 weeks.	VV-SED vs. VV-EXE: KYNA↔ KAT1/2/3 mRNA↑ KMO mRNA↑ KAT1 ↑ KAT2 ↔	MM-SED vs. MM-EXE: KYNA↔ KAT1 mRNA↔ KAT2/4 mRNA↑ KMO mRNA↑ KAT1 ↑ KAT2 ↔	MM-SED vs. VV-SED: KYNA↑ KAT2 ↑ IDO1 mRNA↑ KAT1/3/4 mRNA↔ KMO mRNA↔ MM-EXE vs. VV-EXE: KYNA↑ IDO1 mRNA↑ KAT1/3/4 mRNA↔ KMO mRNA↑ KAT2↑	Hippocampus
(Isung et al. 2021)	Acute exercise group (Acute) (14) Training intervention group (Training) (13)	Acute: Four consecutive days of high-intensity aerobic exercise. Days 1 and 3: Interval running (1 min RPE 18–19 + 2 min RPE 9–11). Days 2 and 4: Continuous running (average RPE 14–16). Training: Continuous running (average RPE 14–16). ≥30 min per session, 3 times per week, for 4 weeks.	Acute: KYNA↑ 3-HK↑ PIC↑ KYN/TRP↔	Training: KYNA↔ 3-HK↔ PIC↔ KYN/TRP↑	Acute vs. Training: TRP KYNA↑ PIC↑	CSF

## 6 KP may mediate the effects of exercise in the prevention and treatment of AD

Studies have shown that immune dysfunction (Liu et al., 2023), Glu excitotoxicity (Wang and Reddy, 2017), and tau phosphorylation (Rawat et al., 2022) are closely associated with the abnormal development of AD., The immune system, glutamatergic system, and protein kinase/phosphatase system involved in these factors interact and regulate each other through the KP. Changes in KP enzyme expression or related metabolic product levels induced by exercise may represent potential therapeutic targets for AD prevention and treatment. Although no studies have reported on the effects of exercise on peripheral and central KP regulation in AD patients, animal studies have confirmed that exercise significantly influences central KP in mouse AD models and significantly improves AD symptoms (Souza et al., 2017). Studies have shown that TRP and KYN can cross the blood-brain barrier, while KYNA and QUIN cannot (Joisten et al., 2021). In the central nervous system, KYN is metabolized by KP into neurotoxic products (such as 3-HK and QUIN) and neuroprotective products (such as KYNA). Therefore, peripheral clearance of TRP and KYN may prevent pathological accumulation of KYN in the central nervous system (Joisten et al., 2020), thereby achieving preventive and therapeutic effects against neurodegenerative diseases (Agudelo et al., 2014).

**Figure 2:**
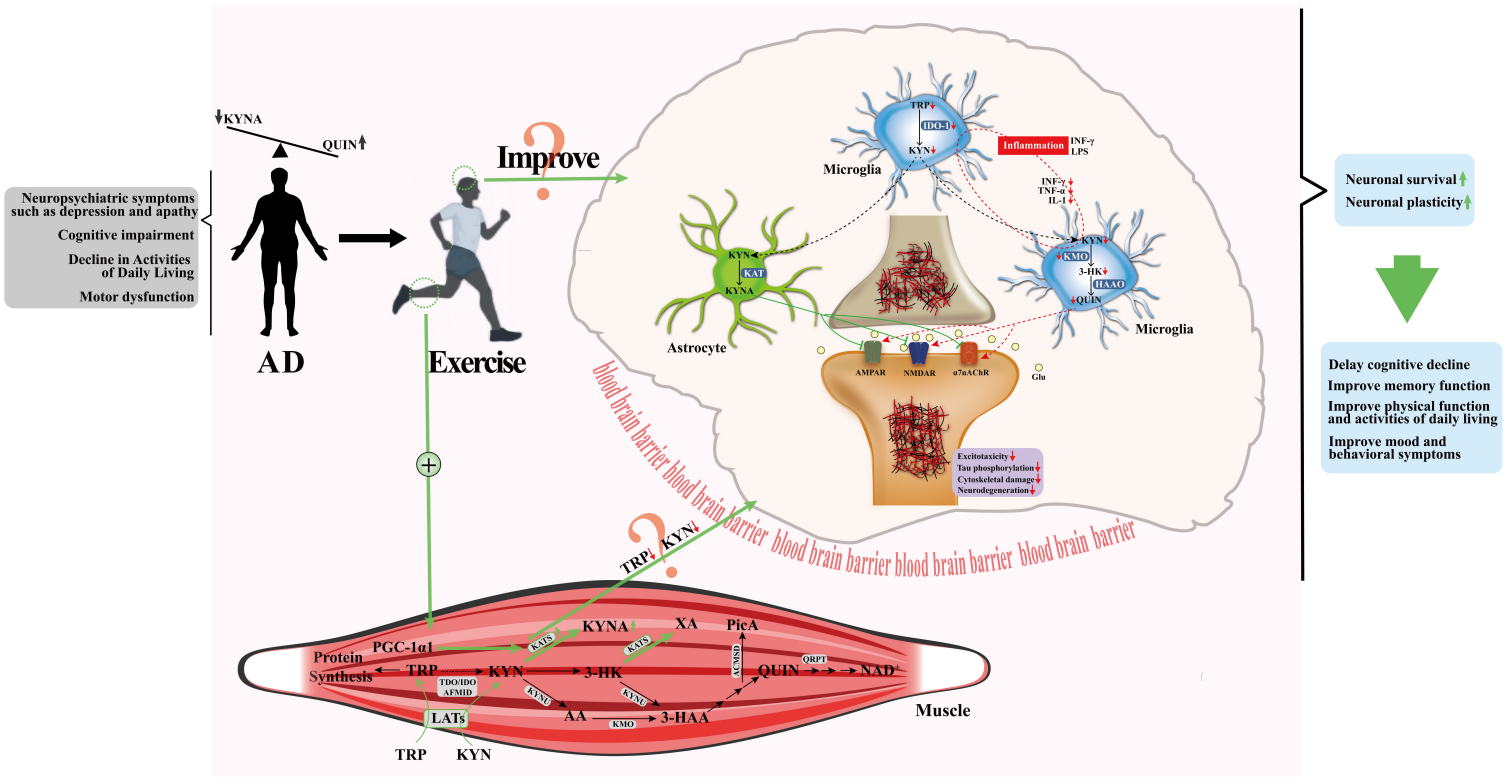
Exercise and KP metabolism in skeletal muscle and central nervous system in AD. Modified from (Martins et al. 2023). **Green arrows** indicate movement status and **red downward arrows** indicate decrease.

Possible mechanisms underlying neuroprotection in exercise-mediated KP have been investigated. Agudelo et al. found that exercise significantly upregulated KATs expression by activating the PGC-1α signaling pathway in skeletal muscle, thereby converting plasma KYN into KYNA that cannot penetrate the blood-brain barrier and reducing its neurotoxic penetration into the central nervous system (Agudelo et al., 2014). In addition, Wang et al. investigated how exercise regulates KP metabolism through the microbiota-gut-brain axis to exert anti-AD effects, emphasizing the central role of gut microbes in KP regulation and the positive effects of exercise on it (Wang et al., 2025). In the context of the above exercise-mediated KP regulation mechanisms, this study further focused on: (1) whether exercise enhances peripheral KYN clearance by activating skeletal muscle KATs, reduces central KP neurotoxic metabolic load, and reverses KP neurotoxicity and neuroprotective imbalance; (2) whether exercise can directly regulate central KP metabolism, predispose it to neuroprotective metabolic branches, promote the production of neuroprotective products (e.g., KYNA), and antagonize toxic products to exert neuroprotective effects (Figure 2). However, the current evidence gap supporting the role of exercise in mediating AD prevention and treatment through KP requires further clarification: (1) The available key evidence is mainly derived from animal models (the majority of rodents), while the direct effects of exercise on KP metabolites and enzyme activities in the periphery and CNS of AD are still lacking to be supported by human study data. (2) Is there an inevitable link between exercise improving AD symptoms and altered KP metabolism? Future human studies are needed to directly validate the association between exercise-induced KP and improvement of symptoms characteristic of AD. (3) Which enzyme in KP plays a dominant role in AD exercise prevention and treatment? This needs to first be verified at the basic level by transgenic animal models combined with optogenetic and/or chemogenetic strategies, specific enzyme agonists and/or inhibitors. On this basis, in order to further confirm the mechanism of KP-mediated AD exercise prevention and treatment, it is also necessary to reveal the spatial association between key KP enzyme (such as IDO, KMO) activity and Aβ and tau pathological deposition in the brain in combination with brain imaging techniques (such as PET, fMRI) in the future, explore the efficacy differences of exercise intervention in achieving AD prevention and treatment by regulating KP, and optimize exercise prescription parameters (exercise intensity, frequency, and type) to achieve individualized AD management.

## 7 Summary and outlook

### 7.1 Summary

(1) KP is a common pathway of action of factors associated with the development of AD (central nervous inflammation, Glu excitotoxicity and tau phosphorylation, etc.). Promoting KP propensity to produce neuroprotective metabolite branches may be a novel therapeutic strategy for AD.
(2) Frequent and regular participation in physical activity is not only significantly associated with a reduced risk of AD, but is also able to significantly improve AD symptoms.
(3) the molecular mechanism by which exercise improves AD symptoms may be related to exercise enhancing the conversion of peripheral KYN to KYNA by activating skeletal muscle KATs on the one hand, decreasing peripheral KYN levels and increasing KYNA levels and protecting the CNS from KYN accumulation; on the other hand, it is related to the generation of neuroprotective neuroactive product branches by predisposing KPs in the CNS. And this still needs to be confirmed by further studies.

In summary, KP is closely related to the occurrence and development of AD, regular exercise may regulate the balance of KP metabolism in the central and/or peripheral, so as to achieve AD exercise prevention and treatment, and this still needs to be confirmed by further studies.

### 7.2 Outlook

Scientific exercise/physical activity is an important component of the public health promotion program for AD (AD health management). However, the neuroprotective effect of exercise intervention in AD and the molecular basis of its pathology and symptom improvement remain to be deeply elucidated. Recent studies have mainly focused on autophagy, oxidative stress, Glu excitotoxicity, mitochondrial dysfunction, cholinergic deletion and other perspectives to explore the possible neurobiological mechanism of exercise in the prevention and treatment of AD. Combined with the above exercise and KP, KP and AD occurrence and development and the role of prevention and treatment and exercise in AD prevention and treatment, it is speculated that KP key metabolic enzymes (IDO, KMO and KAT) may be a new target to mediate AD exercise prevention and treatment. In subsequent studies, we will employ integrated pharmacological modulation and genetic modification strategies. This will include utilizing gene editing technologies (e.g., Cre-LoxP and CRISPR-Cas9) alongside advanced cellular and molecular imaging techniques to achieve cell- and tissue-specific knockdown or knockout of key KP metabolic enzymes. These approaches will elucidate the critical role of KP enzymes in exercise-mediated prevention and mitigation of AD, thereby validating their feasibility as novel therapeutic targets for exercise-based AD interventions. This work will provide essential theoretical foundations and innovative perspectives for research on the neurobiological mechanisms underlying exercise-induced alleviation of AD-related symptoms, as well as for targeted therapeutic strategies.

## References

[B1] Abreu M. Hartley G. (2013). The effects of Salsa dance on balance, gait, and fall risk in a sedentary patient with Alzheimer's dementia, multiple comorbidities, and recurrent falls. J. Geriat. Phys. Ther. 36, 100–108. 10.1519/JPT.0b013e318267aa54 22955042

[B2] Agudelo L. Z. Femenía T. Orhan F. Porsmyr-Palmertz M. Goiny M. Martinez-Redondo V.. (2014). Skeletal muscle PGC-1α1 modulates kynurenine metabolism and mediates resilience to stress-induced depression. Cell 159, 33–45. 10.1016/j.cell.2014.07.051 25259918

[B3] Ahn N. Kim K. (2015). Effects of an elastic band resistance exercise program on lower extremity muscle strength and gait ability in patients with Alzheimer's disease. J. Phys. Ther. Sci. 27, 1953–1955. 10.1589/jpts.27.1953 26180356 PMC4500019

[B4] Allison D. J. Nederveen J. P. Snijders T. Bell K. E. Kumbhare D. Phillips S. M.. (2019). Exercise training impacts skeletal muscle gene expression related to the kynurenine pathway. American journal of physiology. Cell Physiol. 316, C444–C448. 10.1152/ajpcell.00448.2018 30649918 PMC6457098

[B5] Andrade-Guerrero J. Rodríguez-Arellano P. Barron-Leon N. Orta-Salazar E. Ledesma-Alonso C. Díaz-Cintra S.. (2023). Advancing Alzheimer's therapeutics: exploring the impact of physical exercise in animal models and patients. Cells 12:2531. 10.3390/cells12212531 37947609 PMC10648553

[B6] Bakker L. Köhler S. Eussen S. J. P. M. Choe K. van den Hove D. L. A. Kenis G.. (2023). Correlations between kynurenines in plasma and CSF, and their relation to markers of Alzheimer's disease pathology. Brain Behav. Immun. 111, 312–319. 10.1016/j.bbi.2023.04.015 37149106

[B7] Baran H. Jellinger K. Deecke L. (1999). Kynurenine metabolism in Alzheimer's disease. J. Neural Trans. 106, 165–181. 10.1007/s007020050149 10226937

[B8] Ben Ayed I. Ammar A. Aouichaoui C. Mezghani N. Salem A. Naija S.. (2024). Does acute aerobic exercise enhance selective attention, working memory, and problem-solving abilities in Alzheimer's patients? A sex-based comparative study. Front. Sports Active Living 6:1383119. 10.3389/fspor.2024.1383119 38903391 PMC11187274

[B9] Ben Ayed I. Castor-Guyonvarch N. Amimour S. Naija S. Aouichaoui C. Ben Omor S.. (2021). Acute exercise and cognitive function in Alzheimer's disease. J. Alzheimer's Dis. 82, 749–760. 10.3233/JAD-201317 34092631

[B10] Boyle P. A. Buchman A. S. Wilson R. S. Leurgans S. E. Bennett D. A. (2009). Association of muscle strength with the risk of Alzheimer disease and the rate of cognitive decline in community-dwelling older persons. Arch. Neurol. 66, 1339–1344. 10.1001/archneurol.2009.240 19901164 PMC2838435

[B11] Brasure M. Desai P. Davila H. Nelson V. A. Calvert C. Jutkowitz E.. (2018). Physical activity interventions in preventing cognitive decline and Alzheimer-type dementia: a systematic review. Ann. Intern. Med. 168, 30–38. 10.7326/M17-1528 29255839

[B12] Breda C. Sathyasaikumar K. V. Sograte Idrissi S. Notarangelo F. M. Estranero J. G. Moore G. G.. (2016). Tryptophan-2,3-dioxygenase (TDO) inhibition ameliorates neurodegeneration by modulation of kynurenine pathway metabolites. Proc. Natl. Acad. Sci. USA. 113, 5435–5440. 10.1073/pnas.1604453113 27114543 PMC4868470

[B13] Buchman A. S. Wilson R. S. Boyle P. A. Bienias J. L. Bennett D. A. (2007). Grip strength and the risk of incident Alzheimer's disease. Neuroepidemiology 29, 66–73. 10.1159/000109498 17925596

[B14] Chang M. C. Lee A. Y. Kwak S. Kwak S. G. (2020). Effect of resistance exercise on depression in mild Alzheimer disease patients with sarcopenia. Am. J. Geriat. Psychiat. 28, 587–589. 10.1016/j.jagp.2019.07.013 31444046

[B15] Chatterjee P. Zetterberg H. Goozee K. Lim C. K. Jacobs K. R. Ashton N. J.. (2019). Plasma neurofilament light chain and amyloid-β are associated with the kynurenine pathway metabolites in preclinical Alzheimer's disease. J. Neuroinflamm. 16:186. 10.1186/s12974-019-1567-4 31601232 PMC6788092

[B16] Chen P. Geng X. (2023). Research progress on the kynurenine pathway in the prevention and treatment of Parkinson's disease. J. Enzyme Inhib. Med. Chem. 38:2225800. 10.1080/14756366.2023.2225800 37381707 PMC10312032

[B17] Chiesi F. Gori E. Collini F. Palfrader A. Galli R. Guazzini A.. (2021). Biodanza as a nonpharmacological dance movement-based treatment in older people with Alzheimer's disease: an Italian pilot study in 2 tuscan nursing homes. Holist. Nurs. Pract. 35, 264–272. 10.1097/HNP.0000000000000470 34407024

[B18] Colín-González A. L. Maya-López M. Pedraza-Chaverrí J. Ali S. F. Chavarría A. Santamaría A.. (2014). The Janus faces of 3-hydroxykynurenine: dual redox modulatory activity and lack of neurotoxicity in the rat striatum. Brain Res. 1589, 1–14. 10.1016/j.brainres.2014.09.034 25251594

[B19] Cortés Malagón E. M. López Ornelas A. Olvera Gómez I. Bonilla Delgado J. (2024). The Kynurenine pathway, aryl hydrocarbon receptor, and Alzheimer's disease. Brain Sci. 14:950. 10.3390/brainsci14090950 39335444 PMC11429728

[B20] Cummings J. Lee G. Zhong K. Fonseca J. Taghva K. (2021). Alzheimer's disease drug development pipeline: 2021. Alzheimer's Dementia 7:e12179. 10.1002/trc2.12179 34095440 PMC8145448

[B21] De la Rosa A. Olaso-Gonzalez G. Arc-Chagnaud C. Millan F. Salvador-Pascual A. García-Lucerga C.. (2020). Physical exercise in the prevention and treatment of Alzheimer's disease. J. Sport Health Sci. 9, 394–404. 10.1016/j.jshs.2020.01.004 32780691 PMC7498620

[B22] Deora G. S. Kantham S. Chan S. Dighe S. N. Veliyath S. K. McColl G.. (2017). Multifunctional analogs of kynurenic acid for the treatment of Alzheimer's disease: synthesis, pharmacology, and molecular modeling studies. ACS Chem. Neurosci. 8, 2667–2675. 10.1021/acschemneuro.7b00229 28825789

[B23] Enette L. Vogel T. Merle S. Valard-Guiguet A. G. Ozier-Lafontaine N. Neviere R.. (2020). Effect of 9 weeks continuous vs. interval aerobic training on plasma BDNF levels, aerobic fitness, cognitive capacity and quality of life among seniors with mild to moderate Alzheimer's disease: a randomized controlled trial. Eur. Rev. Aging Phys. Activ. 17:2. 10.1186/s11556-019-0234-1 31921371 PMC6945614

[B24] Ferreira F. S. Biasibetti-Brendler H. Pierozan P. Schmitz F. Bertó C. G. Prezzi C. A.. (2018). Kynurenic acid restores Nrf2 levels and prevents quinolinic acid-induced toxicity in rat striatal slices. Mol. Neurobiol. 55, 8538–8549. 10.1007/s12035-018-1003-2 29564809

[B25] Friedland R. P. Fritsch T. Smyth K. A. Koss E. Lerner A. J. Chen C. H.. (2001). Patients with Alzheimer's disease have reduced activities in midlife compared with healthy control-group members. Proc. Natl. Acad. Sci. USA. 98, 3440–3445. 10.1073/pnas.061002998 11248097 PMC30672

[B26] Garuffi M. Costa J. L. Hernández S. S. Vital T. M. Stein A. M. dos Santos J. G.. (2013). Effects of resistance training on the performance of activities of daily living in patients with Alzheimer's disease. Geriatr. Gerontol. Int. 13, 322–328. 10.1111/j.1447-0594.2012.00899.x 22726761

[B27] Giil L. M. Midttun Ø. Refsum H. Ulvik A. Advani R. Smith A. D.. (2017). Kynurenine pathway metabolites in Alzheimer's disease. J. Alzheimer's Dis. 60, 495–504. 10.3233/JAD-170485 28869479

[B28] González-Sánchez M. Jiménez J. Narváez A. Antequera D. Llamas-Velasco S. Martín A. H.. (2020). Kynurenic acid levels are increased in the CSF of Alzheimer's disease patients. Biomolecules 10:571. 10.3390/biom10040571 32276479 PMC7226436

[B29] Guillemin G. J. (2012). Quinolinic acid, the inescapable neurotoxin. FEBS J. 279, 1356–1365. 10.1111/j.1742-4658.2012.08485.x 22248144

[B30] Guillemin G. J. Brew B. J. Noonan C. E. Takikawa O. Cullen K. M. (2005). Indoleamine 2,3 dioxygenase and quinolinic acid immunoreactivity in Alzheimer's disease hippocampus. Neuropathol. Appl. Neurobiol. 31, 395–404. 10.1111/j.1365-2990.2005.00655.x 16008823

[B31] Guillemin G. J. Smythe G. A. Veas L. A. Takikawa O. Brew B. J. (2003). A beta 1-42 induces production of quinolinic acid by human macrophages and microglia. Neuroreport 14, 2311–2315. 10.1097/00001756-200312190-00005 14663182

[B32] Gulaj E. Pawlak K. Bien B. Pawlak D. (2010). Kynurenine and its metabolites in Alzheimer's disease patients. Adv. Med. Sci. 55, 204–211. 10.2478/v10039-010-0023-6 20639188

[B33] Hennings A. Schwarz M. J. Riemer S. Stapf T. M. Selberdinger V. B. Rief W.. (2013). Exercise affects symptom severity but not biological measures in depression and somatization—results on IL-6, neopterin, tryptophan, kynurenine and 5-HIAA. Psychiatry Res. 210, 925–933. 10.1016/j.psychres.2013.09.018 24140252

[B34] Herrstedt A. Bay M. L. Simonsen C. Sundberg A. Egeland C. Thorsen-Streit S.. (2019). Exercise-mediated improvement of depression in patients with gastro-esophageal junction cancer is linked to kynurenine metabolism. Acta Oncologica 58, 579–587. 10.1080/0284186X.2018.1558371 30696326

[B35] Hestad K. Alexander J. Rootwelt H. Aaseth J. O. (2022). The role of tryptophan dysmetabolism and quinolinic acid in depressive and neurodegenerative diseases. Biomolecules 12:998. 10.3390/biom12070998 35883554 PMC9313172

[B36] Holthoff V. A. Marschner K. Scharf M. Steding J. Meyer S. Koch R.. (2015). Effects of physical activity training in patients with Alzheimer's dementia: results of a pilot RCT study. PLoS ONE 10:e0121478. 10.1371/journal.pone.0121478 25884637 PMC4401690

[B37] Hughes T. D. Güner O. F. Iradukunda E. C. Phillips R. S. Bowen J. P. (2022). The Kynurenine Pathway and Kynurenine 3-Monooxygenase Inhibitors. Molecules 27:273. 10.3390/molecules27010273 35011505 PMC8747024

[B38] Hull B. T. Miller K. M. Corban C. Backer G. Sheehan S. Korstanje R.. (2024). 3-hydroxyanthranilic acid delays paralysis in caenorhabditis elegans models of amyloid-beta and polyglutamine proteotoxicity. Biomolecules 14:599. 10.3390/biom14050599 38786006 PMC11117628

[B39] Ieraci A. Beggiato S. Ferraro L. Barbieri S. S. Popoli M. (2020). Kynurenine pathway is altered in BDNF Val66Met knock-in mice: Effect of physical exercise. Brain Behav. Immun. 89, 440–450. 10.1016/j.bbi.2020.07.031 32726686

[B40] Iso-Markku P. Kujala U. M. Knittle K. Polet J. Vuoksimaa E. Waller K.. (2022). Physical activity as a protective factor for dementia and Alzheimer's disease: systematic review, meta-analysis and quality assessment of cohort and case-control studies. Br. J. Sports Med. 56, 701–709. 10.1136/bjsports-2021-104981 35301183 PMC9163715

[B41] Isung J. Granqvist M. Trepci A. Huang J. Schwieler L. Kierkegaard M.. (2021). Differential effects on blood and cerebrospinal fluid immune protein markers and kynurenine pathway metabolites from aerobic physical exercise in healthy subjects. Sci. Rep. 11:1669. 10.1038/s41598-021-81306-4 33462306 PMC7814004

[B42] Jacobs K. R. Lim C. K. Blennow K. Zetterberg H. Chatterjee P. Martins R. N.. (2019). Correlation between plasma and CSF concentrations of kynurenine pathway metabolites in Alzheimer's disease and relationship to amyloid-β and tau. Neurobiol. Aging 80, 11–20. 10.1016/j.neurobiolaging.2019.03.015 31055163

[B43] Jiang B. Feng C. Hu H. George D. Huang T. Li Z.. (2022). Traditional Chinese exercise for neurodegenerative diseases: a bibliometric and visualized analysis with future directions. Front. Aging Neurosci. 14:932924. 10.3389/fnagi.2022.932924 35832067 PMC9271864

[B44] Joisten N. Ruas J. L. Braidy N. Guillemin G. J. Zimmer P. (2021). The kynurenine pathway in chronic diseases: a compensatory mechanism or a driving force? Trends Mol. Med. 27, 946–954. 10.1016/j.molmed.2021.07.006 34373202

[B45] Joisten N. Walzik D. Metcalfe A. J. Bloch W. Zimmer P. (2020). Physical exercise as kynurenine pathway modulator in chronic diseases: implications for immune and energy homeostasis. Int. J. Tryptophan Res. 13:1178646920938688. 10.1177/1178646920938688 32684749 PMC7346690

[B46] Kalyvas A. C. Dimitriou M. Ioannidis P. Grigoriadis N. Afrantou T. (2024). Alzheimer's disease and epilepsy: exploring shared pathways and promising biomarkers for future treatments. J. Clin. Med. 13:3879. 10.3390/jcm13133879 38999445 PMC11242231

[B47] Kaushik M. Yadav A. Upadhyay A. Gupta A. Tiwari P. Tripathi M.. (2025). Yoga an integrated mind body intervention for improvement in quality of life in individuals with Alzheimer's disease and their caregivers. Front. Aging 6:1449485. 10.3389/fragi.2025.1449485 40191145 PMC11968721

[B48] Knapskog A. B. Aksnes M. Edwin T. H. Ueland P. M. Ulvik A. Fang E. F.. (2023). Higher concentrations of kynurenic acid in CSF are associated with the slower clinical progression of Alzheimer's disease. Alzheimer's Dementia 19, 5573–5582. 10.1002/alz.13162 37264981

[B49] Kubicova L. Hadacek F. Weckwerth W. Chobot V. (2015). Effects of endogenous neurotoxin quinolinic acid on reactive oxygen species production by Fenton reaction catalyzed by iron or copper. J. Organomet. Chem. 782, 111–115. 10.1016/j.jorganchem.2015.01.030 25892824 PMC4396856

[B50] Lee D. Y. Im S. C. Kang N. Y. Kim K. (2023). Analysis of effect of intensity of aerobic exercise on cognitive and motor functions and neurotrophic factor expression patterns in an Alzheimer's Disease rat model. J. Pers. Med. 13:1622. 10.3390/jpm13111622 38003937 PMC10672300

[B51] Lewis G. D. Farrell L. Wood M. J. Martinovic M. Arany Z. Rowe G. C.. (2010). Metabolic signatures of exercise in human plasma. Sci. Transl. Med. 2:33ra37. 10.1126/scitranslmed.3001006 20505214 PMC3010398

[B52] Li D. Jia J. Zeng H. Zhong X. Chen H. Yi C.. (2024). Efficacy of exercise rehabilitation for managing patients with Alzheimer's disease. Neural Regen. Res. 19, 2175–2188. 10.4103/1673-5374.391308 38488551 PMC11034587

[B53] Liang Y. Xie S. He Y. Xu M. Qiao X. Zhu Y.. (2022). Kynurenine pathway metabolites as biomarkers in Alzheimer's disease. Dis. Mark. 2022:9484217. 10.1155/2022/9484217 35096208 PMC8791723

[B54] Liu R. Qu H. (2021). Study on the regulation mechanism of aerobic exercise intervention on hippocampal neuroinflammation and improvement of NF-κB, TNF-α/IDO/5-HT signaling pathway in CUMS mice. Chin. J. Immunol. 37, 1563–1570.

[B55] Liu W. Sheng H. Xu Y. Liu Y. Lu J. Ni X. (2013). Swimming exercise ameliorates depression-like behavior in chronically stressed rats: relevant to proinflammatory cytokines and IDO activation. Behav. Brain Res. 242, 110–116. 10.1016/j.bbr.2012.12.041 23291157

[B56] Liu Y. Tan Y. Zhang Z. Li H. Yi M. Zhang Z.. (2023). Neuroimmune mechanisms underlying Alzheimer's disease: insights into central and peripheral immune cell crosstalk. Ageing Res. Rev. 84:101831. 10.1016/j.arr.2022.101831 36565960

[B57] Lovelace M. D. Varney B. Sundaram G. Franco N. F. Ng M. L. Pai S.. (2016). Current evidence for a role of the kynurenine pathway of tryptophan metabolism in multiple sclerosis. Front. Immunol. 7:246. 10.3389/fimmu.2016.00246 27540379 PMC4972824

[B58] Lugo-Huitrón R. Blanco-Ayala T. Ugalde-Muñiz P. Carrillo-Mora P. Pedraza-Chaverrí J. Silva-Adaya D.. (2011). On the antioxidant properties of kynurenic acid: free radical scavenging activity and inhibition of oxidative stress. Neurotoxicol. Teratol. 33, 538–547. 10.1016/j.ntt.2011.07.002 21763768

[B59] Lugo-Huitrón R. Ugalde Muñiz P. Pineda B. Pedraza-Chaverrí J. Ríos C. Pérez-de la Cruz V. (2013). Quinolinic acid: an endogenous neurotoxin with multiple targets. Oxid. Med. Cell. Longev. 2013:104024. 10.1155/2013/104024 24089628 PMC3780648

[B60] Majerova P. Olesova D. Golisova G. Buralova M. Michalicova A. Vegh J.. (2022). Analog of kynurenic acid decreases tau pathology by modulating astrogliosis in rat model for tauopathy. Biomed. Pharmacother. 152:113257. 10.1016/j.biopha.2022.113257 35714514

[B61] Marques-Aleixo I. Oliveira P. J. Moreira P. I. Magalhães J. Ascensão A. (2012). Physical exercise as a possible strategy for brain protection: evidence from mitochondrial-mediated mechanisms. Progr. Neurobiol. 99, 149–162. 10.1016/j.pneurobio.2012.08.002 22940590

[B62] Martin K. S. Azzolini M. Lira Ruas J. (2020). The kynurenine connection: how exercise shifts muscle tryptophan metabolism and affects energy homeostasis, the immune system, and the brain. Am. J. Physiol. Cell Physiol. 318, C818–C830. 10.1152/ajpcell.00580.2019 32208989

[B63] Martins L. B. Silveira A. L. M. Teixeira A. L. (2023). The involvement of kynurenine pathway in neurodegenerative diseases. Curr. Neuropharmacol. 21, 260–272. 10.2174/1570159X20666220922153221 36154606 PMC10190152

[B64] Mieszkowski J. Brzezińska P. Stankiewicz B. Kochanowicz A. Niespodziński B. Reczkowicz J.. (2022). Direct effects of vitamin D supplementation on ultramarathon-induced changes in kynurenine metabolism. Nutrients 14:4485. 10.3390/nu14214485 36364748 PMC9655671

[B65] Minhas P. S. Jones J. R. Latif-Hernandez A. Sugiura Y. Durairaj A. S. Wang Q.. (2024). Restoring hippocampal glucose metabolism rescues cognition across Alzheimer's disease pathologies. Science 385:eabm6131. 10.1126/science.abm6131 39172838 PMC12313320

[B66] Mithaiwala M. N. Santana-Coelho D. Porter G. A. O'Connor J. C. (2021). Neuroinflammation and the kynurenine pathway in cns disease: molecular mechanisms and therapeutic implications. Cells 10:1548. 10.3390/cells10061548 34205235 PMC8235708

[B67] Mor A. Tankiewicz-Kwedlo A. Krupa A. Pawlak D. (2021). Role of kynurenine pathway in oxidative stress during neurodegenerative disorders. Cells 10:1603. 10.3390/cells10071603 34206739 PMC8306609

[B68] Mudry J. M. Alm P. S. Erhardt S. Goiny M. Fritz T. Caidahl K.. (2016). Direct effects of exercise on kynurenine metabolism in people with normal glucose tolerance or type 2 diabetes. Diabetes Metab. Res. Rev. 32, 754–761. 10.1002/dmrr.2798 26946084

[B69] Pal A. Zimmer P. Clauss D. Schmidt M. E. Ulrich C. M. Wiskemann J.. (2021). Resistance exercise modulates kynurenine pathway in pancreatic cancer patients. Int. J. Sports Med. 42, 33–40. 10.1055/a-1186-1009 32707579

[B70] Papatsimpas V. Vrouva S. Papathanasiou G. Papadopoulou M. Bouzineki C. Kanellopoulou S.. (2023). Does therapeutic exercise support improvement in cognitive function and instrumental activities of daily living in patients with mild Alzheimer's Disease? A randomized controlled trial. Brain Sci. 13:1112. 10.3390/brainsci13071112 37509042 PMC10377697

[B71] Parker D. C. Kraus W. E. Whitson H. E. Kraus V. B. Smith P. J. Cohen H. J.. (2023). Tryptophan metabolism and neurodegeneration: longitudinal associations of kynurenine pathway metabolites with cognitive performance and plasma Alzheimer's disease and related dementias biomarkers in the duke physical performance across the lifespan study. J. Alzheimer's Dis. 91, 1141–1150. 10.3233/JAD-220906 36565121 PMC10074831

[B72] Pérez-De La Cruz V. Carrillo-Mora P. Santamaría A. (2012). Quinolinic acid, an endogenous molecule combining excitotoxicity, oxidative stress and other toxic mechanisms. Int. J. Tryptophan Res. 5, 1–8. 10.4137/IJTR.S8158 22408367 PMC3296489

[B73] Pierozan P. Biasibetti-Brendler H. Schmitz F. Ferreira F. Pessoa-Pureur R. Wyse A. T. S.. (2018). Kynurenic acid prevents cytoskeletal disorganization induced by quinolinic acid in mixed cultures of rat striatum. Mol. Neurobiol. 55, 5111–5124. 10.1007/s12035-017-0749-2 28840509

[B74] Pierozan P. Zamoner A. Soska A. K. Silvestrin R. B. Loureiro S. O. Heimfarth L.. (2010). Acute intrastriatal administration of quinolinic acid provokes hyperphosphorylation of cytoskeletal intermediate filament proteins in astrocytes and neurons of rats. Exp. Neurol. 224, 188–196. 10.1016/j.expneurol.2010.03.009 20303347

[B75] Platten M. Nollen E. A. A. Röhrig U. F. Fallarino F. Opitz C. A. (2019). Tryptophan metabolism as a common therapeutic target in cancer, neurodegeneration and beyond. Nature reviews. Drug Discov. 18, 379–401. 10.1038/s41573-019-0016-5 30760888

[B76] Pocivavsek A. Schwarcz R. Erhardt S. (2024). Neuroactive kynurenines as pharmacological targets: new experimental tools and exciting therapeutic opportunities. Pharmacol. Rev. 76, 978–1008. 10.1124/pharmrev.124.000239 39304346 PMC11549936

[B77] Puigarnau S. Fernàndez A. Obis E. Jové M. Castañer M. Pamplona R.. (2022). Metabolomics reveals that fittest trail runners show a better adaptation of bioenergetic pathways. J. Sci. Med. Sport 25, 425–431. 10.1016/j.jsams.2021.12.006 35063356

[B78] Rahman A. Ting K. Cullen K. M. Braidy N. Brew B. J. Guillemin G. J.. (2009). The excitotoxin quinolinic acid induces tau phosphorylation in human neurons. PLoS ONE 4:e6344. 10.1371/journal.pone.0006344 19623258 PMC2709912

[B79] Ramachandran A. K. Das S. Joseph A. Shenoy G. G. Alex A. T. Mudgal J. (2021). Neurodegenerative pathways in Alzheimer's Disease: a review. Curr. Neuropharmacol. 19, 679–692. 10.2174/1570159X18666200807130637 32851951 PMC8573750

[B80] Ramos-Chávez L. A. Lugo Huitrón R. González Esquivel D. Pineda B. Ríos C. Silva-Adaya D.. (2018). Relevance of alternative routes of kynurenic acid production in the brain. Oxid. Med. Cell. Longev. 2018:5272741. 10.1155/2018/5272741 29977455 PMC5994304

[B81] Rangel M. V. D. S. Lopes K. G. Qin X. Borges J. P. (2025). Exercise-induced adaptations in the kynurenine pathway: implications for health and disease management. Front. Sports Active Living 7:1535152. 10.3389/fspor.2025.1535152 40115437 PMC11922725

[B82] Rawat P. Sehar U. Bisht J. Selman A. Culberson J. Reddy P. H. (2022). Phosphorylated tau in Alzheimer's Disease and other tauopathies. Int. J. Mol. Sci. 23:12841. 10.3390/ijms232112841 36361631 PMC9654278

[B83] Reddi Sree R. Kalyan M. Anand N. Mani S. Gorantla V. R. Sakharkar M. K.. (2025). Newer therapeutic approaches in treating Alzheimer's disease: a comprehensive review. ACS Omega 10, 5148–5171. 10.1021/acsomega.4c05527 39989768 PMC11840625

[B84] Safiri S. Ghaffari Jolfayi A. Fazlollahi A. Morsali S. Sarkesh A. Daei Sorkhabi A.. (2024). Alzheimer's disease: a comprehensive review of epidemiology, risk factors, symptoms diagnosis, management, caregiving, advanced treatments and associated challenges. Front. Med. 11:1474043. 10.3389/fmed.2024.1474043 39736972 PMC11682909

[B85] Sánchez Chapul L. de Pérez G. la Cruz L. A. Ramos Chávez J. F. Valencia León J. Torres Beltrán E.. (2022). Characterization of redox environment and tryptophan catabolism through kynurenine pathway in military divers' and swimmers' serum samples. Antioxidants 11:1223. 10.3390/antiox11071223 35883715 PMC9312203

[B86] Savonije K. Weaver D. F. (2023). The role of tryptophan metabolism in Alzheimer's disease. Brain Sci. 13:292. 10.3390/brainsci13020292 36831835 PMC9954102

[B87] Schlittler M. Goiny M. Agudelo L. Z. Venckunas T. Brazaitis M. Skurvydas A.. (2016). Endurance exercise increases skeletal muscle kynurenine aminotransferases and plasma kynurenic acid in humans. Am. J. Physiol. Cell Physiol. 310, C836–C840. 10.1152/ajpcell.00053.2016 27030575

[B88] Sepúlveda-Lara A. Sepúlveda P. Marzuca-Nassr G. N. (2024). Resistance exercise training as a new trend in Alzheimer's disease research: from molecular mechanisms to prevention. Int. J. Mol. Sci. 25:7084. 10.3390/ijms25137084 39000191 PMC11241132

[B89] Sorgdrager F. J. H. Vermeiren Y. Van Faassen M. van der Ley C. Nollen E. A. A. Kema I. P.. (2019). Age- and disease-specific changes of the kynurenine pathway in Parkinson's and Alzheimer's disease. J. Neurochem. 151, 656–668. 10.1111/jnc.14843 31376341 PMC6899862

[B90] Souza L. C. Jesse C. R. Antunes M. S. Ruff J. R. de Oliveira Espinosa D. Gomes N. S.. (2016). Indoleamine-2,3-dioxygenase mediates neurobehavioral alterations induced by an intracerebroventricular injection of amyloid-β1-42 peptide in mice. Brain Behav. Immun. 56, 363–377. 10.1016/j.bbi.2016.03.002 26965653

[B91] Souza L. C. Jesse C. R. Del Fabbro L. de Gomes M. G. Goes A. T. R. Filho C. B.. (2017). Swimming exercise prevents behavioural disturbances induced by an intracerebroventricular injection of amyloid-β1-42 peptide through modulation of cytokine/NF-kappaB pathway and indoleamine-2,3-dioxygenase in mouse brain. Behav. Brain Res. 331, 1–13. 10.1016/j.bbr.2017.05.024 28502732

[B92] Tai S. Y. Hsu C. L. Huang S. W. Ma T. C. Hsieh W. C. Yang Y. H.. (2016). Effects of multiple training modalities in patients with Alzheimer's disease: a pilot study. Neuropsychiatr. Dis. Treat. 12, 2843–2849. 10.2147/NDT.S116257 27843319 PMC5098772

[B93] Tao D. Awan-Scully R. Ash G. I. Pei Z. Gu Y. Gao Y.. (2023). The effectiveness of dance movement interventions for older adults with mild cognitive impairment, Alzheimer's disease, and dementia: a systematic scoping review and meta-analysis. Ageing Res. Rev. 92:102120. 10.1016/j.arr.2023.102120 37944706 PMC11262040

[B94] Tavares R. G. Tasca C. I. Santos C. E. Alves L. B. Porciúncula L. O. Emanuelli T.. (2002). Quinolinic acid stimulates synaptosomal glutamate release and inhibits glutamate uptake into astrocytes. Neurochem. Int. 40, 621–627. 10.1016/S0197-0186(01)00133-4 11900857

[B95] Ting K. K. Brew B. J. Guillemin G. J. (2009). Effect of quinolinic acid on human astrocytes morphology and functions: implications in Alzheimer's disease. J. Neuroinflammation 6:36. 10.1186/1742-2094-6-36 20003262 PMC2797503

[B96] Török N. Tanaka M. Vécsei L. (2020). Searching for peripheral biomarkers in neurodegenerative diseases: the tryptophan-kynurenine metabolic pathway. Int. J. Mol. Sci. 21:9338. 10.3390/ijms21249338 33302404 PMC7762583

[B97] van der Velpen V. Teav T. Gallart-Ayala H. Mehl F. Konz I. Clark C.. (2019). Systemic and central nervous system metabolic alterations in Alzheimer's disease. Alzheimer's Res. Ther. 11:93. 10.1186/s13195-019-0551-7 31779690 PMC6883620

[B98] Vécsei L. Szalárdy L. Fülöp F. Toldi J. (2013). Kynurenines in the CNS: recent advances and new questions. Nature reviews. Drug Discov. 12, 64–82. 10.1038/nrd3793 23237916

[B99] Venturelli M. Scarsini R. Schena F. (2011). Six-month walking program changes cognitive and ADL performance in patients with Alzheimer. Am. J. Alzheimer's Dis. Dementias 26, 381–388. 10.1177/1533317511418956 21852281 PMC10845333

[B100] Vidoni E. D. Perales J. Alshehri M. Giles A. M. Siengsukon C. F. Burns J. M.. (2019). Aerobic exercise sustains performance of instrumental activities of daily living in early-stage Alzheimer disease. J. Geriat. Phys. Ther. 42, E129–E134. 10.1519/JPT.0000000000000172 29286983 PMC6023779

[B101] Vints W. A. J. Gökçe E. Šeikinaite J. Kušleikiene S. Cesnaitiene V. J. Verbunt J.. (2024). Resistance training's impact on blood biomarkers and cognitive function in older adults with low and high risk of mild cognitive impairment: a randomized controlled trial. Eur. Rev. Aging Phys. Activ. 21:9. 10.1186/s11556-024-00344-9 38600451 PMC11005144

[B102] Vital T. M. Hernández S. S. S. Pedroso R. V. Teixeira C. V. L. Garuffi M. Stein A. M.. (2012). Effects of weight training on cognitive functions in elderly with Alzheimer's disease. Dementia Neuropsychologia 6, 253–259. 10.1590/S1980-57642012DN06040009 29213805 PMC5619337

[B103] Walczak K. Gerkowicz A. Krasowska D. (2023). PPARs and the kynurenine pathway in melanoma-potential biological interactions. Int. J. Mol. Sci. 24:3114. 10.3390/ijms24043114 36834531 PMC9960262

[B104] Wang R. Reddy P. H. (2017). Role of Glutamate and NMDA Receptors in Alzheimer's Disease. J. Alzheimer's Dis. 57, 1041–1048. 10.3233/JAD-160763 27662322 PMC5791143

[B105] Wang Y. Zhang Y. Wang W. Zhang Y. Dong X. Liu Y.. (2025). Diverse physiological roles of kynurenine pathway metabolites: updated implications for health and disease. Metabolites 15:210. 10.3390/metabo15030210 40137174 PMC11943880

[B106] Wei Y. H. Jian H. L. Huang X. X. Ye J. R. Yang H. Lu X. L.. (2024). Effects of Baduanjin-based aerobic exercise on cognitive function and quality of life in patients with mild-to-moderate Alzheimer's disease. Evid.-Based Nurs. 10, 335–338.

[B107] Wu H. Q. Lee S. C. Schwarcz R. (2000). Systemic administration of 4-chlorokynurenine prevents quinolinate neurotoxicity in the rat hippocampus. Eur. J. Pharmacol. 390, 267–274. 10.1016/S0014-2999(00)00024-8 10708733

[B108] Wu W. Nicolazzo J. A. Wen L. Chung R. Stankovic R. Bao S. S.. (2013). Expression of tryptophan 2,3-dioxygenase and production of kynurenine pathway metabolites in triple transgenic mice and human Alzheimer's disease brain. PLoS ONE 8:e59749. 10.1371/journal.pone.0059749 23630570 PMC3632609

[B109] Wyckelsma V. L. Trepci A. Schwieler L. Venckunas T. Brazaitis M. Kamandulis S.. (2021). Vitamin C and E treatment blocks changes in kynurenine metabolism triggered by three weeks of sprint interval training in recreationally active elderly humans. Antioxidants 10:1443. 10.3390/antiox10091443 34573075 PMC8465740

[B110] Xiao L. Liu C. Ouyang S. Li Y. Guo Z. Wang A. (2017). The effect of isokinetic strength rehabilitation training on cognitive and motor functions in patients with Alzheimer's disease. Prog. Mod. Biomed. 17, 4330–4333.

[B111] Xue C. Li G. Zheng Q. Gu X. Shi Q. Su Y.. (2023). Tryptophan metabolism in health and disease. Cell Metab. 35, 1304–1326. 10.1016/j.cmet.2023.06.004 37352864

[B112] Yang Y. Liu X. Liu X. Xie C. Shi J. (2024). The role of the kynurenine pathway in cardiovascular disease. Front. Cardiovasc. Med. 11:1406856. 10.3389/fcvm.2024.1406856 38883986 PMC11176437

[B113] Yao L. Giordani B. J. Algase D. L. You M. Alexander N. B. (2013). Fall risk-relevant functional mobility outcomes in dementia following dyadic tai chi exercise. West. J. Nurs. Res. 35, 281–296. 10.1177/0193945912443319 22517441 PMC3468653

[B114] Yu D. Tao B. B. Yang Y. Y. Du L. S. Yang S. S. He X. J.. (2015). The IDO inhibitor coptisine ameliorates cognitive impairment in a mouse model of Alzheimer's disease. J. Alzheimer's Dis. 43, 291–302. 10.3233/JAD-140414 25079795

[B115] Yu F. Vock D. M. Zhang L. Salisbury D. Nelson N. W. Chow L. S.. (2021). Cognitive effects of aerobic exercise in Alzheimer's disease: a pilot randomized controlled trial. J. Alzheimer's Dis. 80, 233–244. 10.3233/JAD-201100 33523004 PMC8075384

[B116] Zádori D. Veres G. Szalárdy L. Klivényi P. Vécsei L. (2018). Alzheimer's disease: recent concepts on the relation of mitochondrial disturbances, excitotoxicity, neuroinflammation, and kynurenines. J. Alzheimer's Dis. 62, 523–547. 10.3233/JAD-170929 29480191

[B117] Zhong M. Z. Peng T. Duarte M. L. Wang M. Cai D. (2024). Updates on mouse models of Alzheimer's disease. Mol. Neurodegener. 19:23. 10.1186/s13024-024-00712-0 38462606 PMC10926682

[B118] Zwilling D. Huang S. Y. Sathyasaikumar K. V. Notarangelo F. M. Guidetti P. Wu H. Q.. (2011). Kynurenine 3-monooxygenase inhibition in blood ameliorates neurodegeneration. Cell 145, 863–868. 10.1016/j.cell.2011.05.020 21640374 PMC3118409

